# Can the Immune System Perform a t-Test?

**DOI:** 10.1371/journal.pone.0169464

**Published:** 2017-01-03

**Authors:** Bruno Filipe Faria, Patricia Mostardinha, Fernao Vistulo de Abreu

**Affiliations:** 1 Department of Physics, University of Aveiro, Aveiro, Portugal; 2 Institute for Biomedicine - iBiMED, University of Aveiro, Aveiro, Portugal; 3 I3N Institute for Nanostructures, Nanomodelling and Nanofabrication, Aveiro, Portugal; Duke University School of Medicine, UNITED STATES

## Abstract

The self-nonself discrimination hypothesis remains a landmark concept in immunology. It proposes that tolerance breaks down in the presence of nonself antigens. In strike contrast, in statistics, occurrence of nonself elements in a sample (i.e., outliers) is not obligatory to violate the null hypothesis. Very often, what is crucial is the combination of (self) elements in a sample. The two views on how to detect a change seem challengingly different and it could seem difficult to conceive how immunological cellular interactions could trigger responses with a precision comparable to some statistical tests. Here it is shown that frustrated cellular interactions reconcile the two views within a plausible immunological setting. It is proposed that the adaptive immune system can be promptly activated either when nonself ligands are detected or self-ligands occur in abnormal combinations. In particular we show that cellular populations behaving in this way could perform location statistical tests, with performances comparable to t or KS tests, or even more general data mining tests such as support vector machines or random forests. In more general terms, this work claims that plausible immunological models should provide accurate detection mechanisms for host protection and, furthermore, that investigation on mechanisms leading to improved detection in “in silico” models can help unveil how the real immune system works.

## Background

It has long been debated whether the main function driving the adaptive immune system is related to its ability to maintain homeostasis [1, 2] or to eliminate foreign substances [3, 4]. On theoretical grounds it has been easier to build models that perform some level of self-nonself discrimination [5–7], even if it has been recognized that perfect self-nonself discrimination could be difficult to achieve [8, 9].

Instead of being focused in detecting foreignness, Niels Jerne and followers [10–12] proposed that the adaptive immune system would be concerned in maintaining homeostasis. Theoretical studies to support these ideas revolved around models of idiotypic networks, whose relevance however proved difficult to demonstrate in practice [13, 14]. After a remarkable initial growth in the 80’s, these ideas were progressively abandoned afterwards [13]. This drawback does not necessarily disprove Niels Jerne main conceptual insights, which are more general than the specific model adopted to test them. According to Jerne, immune interactions should be concerned in maintaining a regulated dynamics with itself. Foreignness could not be associated to specific antigen, but rather emerge from a perturbation in the dynamics.

The absence of a self/nonself discrimination mechanism in Jerne’s conceptual model was the main source of rejection for these ideas. In an attempt to reconcile the two views, Coutinho and Varela proposed a second generation of immune networks [15]. Varela and Coutinho developed extensive numerical work and showed that lymphocytes would be arranged in a giant connected component of self-reactive elements which would be responsible for maintaining homeostasis, plus a set of disconnected peripheral and nonself reactive clones, responsible for eliminating nonself invaders. Today, these directions are still explored with new formalisms, techniques and ideas [16, 17].

Unfortunately, none of the proposed models gained indisputable acceptance being still unclear which proposal provides an effective defence mechanism [18–21]. In this respect it is important to outline the efforts played by a growing community of computing oriented researchers who have been looking into the immune system for inspiration to build better computational algorithms. Indeed, if the immune system is competent in protecting the host from invaders, new computational algorithms could use similar strategies to detect deviations from normal functioning. Considerable high quality theoretical work has been done on different formulations, such as self nonself discrimination models [6, 8], idiotypic networks [16, 17], clonal expansion models [22, 23], and models following the danger hypothesis [24]. The number of applications studied have also been impressive. It could range from fault [25, 26] and intrusion [27–29] detection to mathematical optimization [30–32] and robot path planning [33, 34]. Despite these advances, some have raised doubts on their relevance as compared to those accomplished in fields like artificial intelligence [35].

In any case, the artificial intelligence perspective has two important merits. On one side, it highlights that it is worth studying cellular processes that can encompass accurate anomaly detection, as this is likely to play a role in host protection. On the other side, it defines a research framework for testing the performance of competing theories proposing alternative mechanisms of immune protection. Indeed, our point of view is that the adaptive immune system should work as a sophisticated statistical (or data mining) detector, signalling immune responses whenever deviations from a normal state are detected. This would be analogous to the violation of the null hypothesis in statistical testing.

## The Adaptive Immune System: a Modelling Perspective

Even having three different lineages (Th_1_, Th_2_ and CTL), all T cells from the different subsets take part in a similar complex dynamics of cellular interactions with antigen presenting cells (APCs) (dendritic cells and macrophages) in lymph nodes. In particular, they all require stable contacts [36, 37] and two signals to become activated [38, 39]. The first signal involves interactions of T cell receptors with MHC molecules and a peptide fragment on APCs. The second signal is non-specific and typically involves CD28 and B7 molecules on T cells and APCs, respectively.

T cells play a central role in the adaptive immune system because, upon activation T cells proliferate and differentiate onto: Th_1_ cells, which migrate to sites of infection, where they activate phagocytes that captured microbes with fragments like those detected by the T cell in the lymph node; Th_2_ cells, which can undergo a similar type of complex dynamics with activated B cells, triggering their differentiation into plasma cells and antibody production; cytotoxic T lymphocytes (CTLs), which migrate to sites of infection and eliminate cells harbouring intracellular microbes (like viruses).

Given the central role played by T cells in initiating the immune response, the cellular frustration approach focused on modelling the interaction of APCs and T cells. The strategy has been that if plausible cellular mechanisms would be identified leading to an accurate triggering of immune responses, then this research could enlighten on the role of the different cells and signals exchanged, and what type of information the immune system senses and responds to. Indeed, it is unlikely that an inaccurate initiation of immune responses could lead to effective host protection.

To achieve flexible immunity, the immune system uses somatic recombination to build very diverse receptors, and therefore receptors are not transmitted to the organism’s offspring. To accurately discriminate healthy from non-healthy cells and molecules, the adaptive immune system has to undergo a repertoire education (maturation) stage, which takes place in early life in the thymus, for T cells, and in bone marrow for B cells. Therefore, to establish viable immune protection mechanisms it is crucial to consider the two important stages in T cells’ lifes, the education stage where T cells are selected to recognize displayed antigen and simultaneously they are prevented from reacting against host cells, and the activation stage taking place in specialized organs in the periphery (like the lymph nodes). In both cases, cells should interact following a similar dynamics, since one stage prepares the other.

The cellular frustration framework (CFF) makes an important assumption on the scale of the fundamental processes involved in the definition of the *self*. The CFF assumes that the *self* is a systemic entity and consequently, only mechanisms processing information arising from all constituents simultaneously are likely to conveniently model the detection mechanisms involved in host protection by the adaptive immune system. This has an important modelling consequence, because it assumes that studying how individual cells interact is not enough to define the *self* information. In fact, one the most important results in this article is the demonstration of how context dependent detection mechanisms can be built to capture systemic information, i.e., information on population properties.

To gain access to systemic information, the cellular frustration dynamics assumes that both, T cells and APCs, continuously monitor signals delivered by the cells they contact with and direct their immunological synapses towards the cell delivering the strongest signals. Experimentally it was already demonstrated that T cells can perform cellular decisions of this type(see [40] and in particular the supplementary video 4). Here we will assume that, given the extremely packed environments in the thymus and lymph nodes [41], cellular decisions are continuously taking place by both, APCs and T cells. The other assumption used is that only long contacts allow immunological synapses to mature and trigger effector functions [36, 40]. Hence, instead of being concerned with describing which cells interact with strong avidity, the cellular frustration approach [42] is concerned with which cells establish stable contacts (i.e., long-lived interactions). Furthermore, it will be the increase in the number of long long-lived interactions that will signal the degree of pathogenicity.

In this paper we will show that if one accepts the cellular frustration description of cellular interactions in the adaptive immune system, then the immune system should be capable of signalling the abnormal presentation of peptides with accuracies comparable to well-known statistical tests such as the t-test, or the KS test. This result shows how cellular interactions can aggregate information distributed over many different APCs. So far, quorum sensing is the best known biological mechanism capturing global information on a system’s configuration. In contrast to quorum sensing mechanisms which are sensitive to population frequencies, cellular frustration captures information of the joint frequency of several peptides in the population, and hence it aggregates more information.

## Cellular Frustration Framework as an Unstable Matching Problem

The cellular frustration approach to the adaptive immune system received inspiration from a well-known problem in computational mathematics [43], the stable marriage problem (SMP). This problem was first proposed by Gale and Shapley in 1962 [44]. Due to its relevance to market creation, work in this area was awarded the 2012 Nobel Prize in Economics. The SMP found several applications such as in organ transplant allocation [45] or management of communication networks [46].

In the stable marriage problem, researchers look for efficient algorithms matching men and women in stable pairs [44, 47]. Finding stable solutions—i.e., arrangements with only stable pairs—can be difficult because men and women all have different and complex preferences which can interfere with each other. As a result, some instances of the problem can be NP complex [47], which means that in these cases there is no known deterministic algorithm that finds a stable solution in a reasonable computational time. In any case, even for instances for which efficient algorithms exist, they are not likely to be relevant in the context of cellular immunological interactions, since they require that cells follow a precise sequence of interactions simultaneously.

The cellular frustration approach to the adaptive immune system uses the Gale and Shapley original model as a starting point. Two cell types, T cells and APCs, play the role of men and women. The crucial difference between the two models lies on their aim. The stable marriage problem looks for stable matchings because it is argued that men and women lose time when they engage in unstable matchings. By contrast the cellular frustration framework (CFF) tries to find a subset of T cells engaging in unstable (frustrated) interactions with APCs. Since effector functions require forming stable conjugations (stable pairs), in highly frustrated populations cells are rarely activated despite of their natural tendency to interact. As a result, in CFSs cellular activation is only triggered either in physiologically tolerable numbers or when the dynamics is disrupted due to changes in the information presented by APCs to T cells. Here it will be shown that, besides responses to nonself ligands (see [48]), T cells can be activated if a combination of self-ligands deviates from their typical frequencies of appearance. This is a type of anomaly response that does not require the presence of nonself ligands and for this reason we call it detection of abnormal-self.

**Fig 1:**
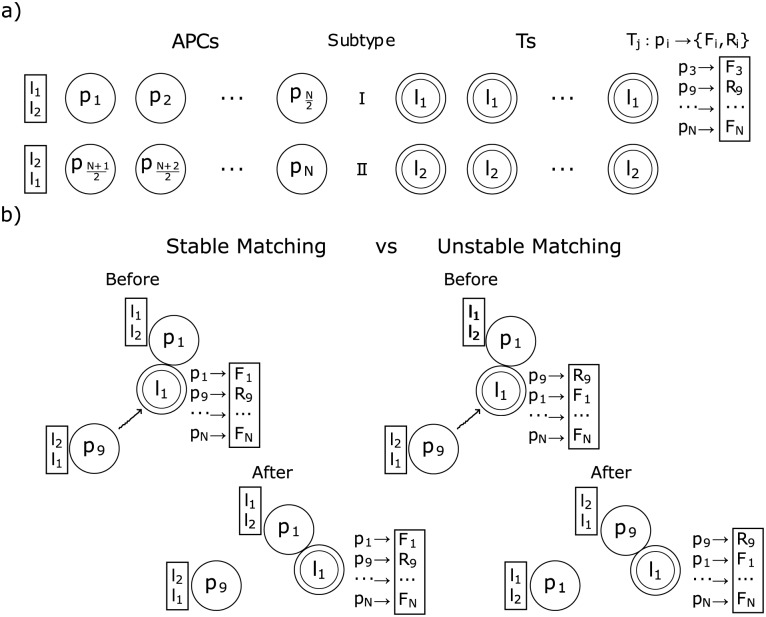
Cellular Frustration model used in this article. a) The model consists of 2*N* cells equally divided in two types, APCs and T cells. Each cell type is further divided in two subtypes, I and II, of the same size, and present ligands to cells of the opposite type. APCs present a diverse set of ligands *p*_*i*_ ($p_{i}\in R$) while T cells present only ligands *l*_1_ or *l*_2_. Ligands *l*_1_ or *l*_2_ determine the T cell subtype and also the APCs subtype, since APCs of subtype I (II) rank ligands *l*_1_ (*l*_2_) first. In this paper it is assumed that T cells map ligands *p*_*i*_ in two ligands (or signals), one presented frequently *F*_*i*_ and the other, *R*_*i*_, only rarely. b) Examples of decisions taken by cells upon interaction. A new matching is formed whenever the displayed ligands are ranked higher in each other’s ILists. This happens in the case on the right but not in the one on the left. In the case on the right the APC displaying the *p*_*q*_ ligand is said to frustrate the interaction between the APC and T cell displaying ligands *p*_1_ and *l*_1_, respectively.

As in the SMP, in cellular frustrated models it is assumed that APCs and T cells have preference lists, named interaction lists (ILists). Following [48], it is assumed that APCs discriminate only the presence of either one of two ligands on T cells, and consequently T cells can be grouped in two cell subtypes. Likewise, APCs can be grouped in two cell subtypes depending on which ligand they rank first. Therefore, in the model studied in this article, there are two cell types and two cell subtypes, as depicted in Fig 1. For simplicity it is also assumed that all cell subtypes have *N*/2 cells.

In the CFF it is assumed that the information displayed on cells’ surfaces can be mapped onto a single ligand. APCs can display a large diversity of possible ligands. On the other hand, T cells display only 2 possible ligands, which is used to define the T cell subtype. APCs subtype is also defined using these 2 ligands as it is assumed that APCs prioritize interactions with T cells of the same subtype.

The typical complex dynamics that can emerge in the SMP arises in cellular frustrated populations as well, because of the complex organization of the ligands displayed by APCs in T cells ILists. In particular, if a T cell would rank in the top position the ligand displayed by an APC of the same cell subtype, then a conjugation between the two cells would be maximally stable. By contrast, if all ligands displayed by APCs of the opposite cell subtype would be ranked first then conjugations would be short lived because even when the T cell is conjugated to the ligand ranked in the first position, all APCs of the opposite cell subtype can destabilize the conjugation. Cells destabilizing conjugations are said to frustrate interactions. The cellular frustration framework puts a special emphasis on the importance of frustration to organize the dynamics and perform accurate intrusion detection, as discussed next.

## Ordering and Detection in Cellular Frustrated Populations

**Fig 2:**
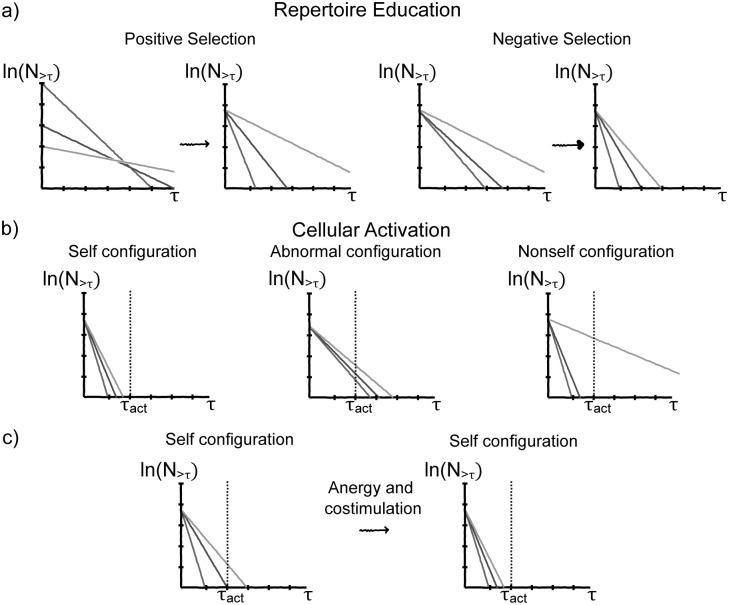
Important immunological mechanisms and their role according to the CFF. The number of conjugations lasting for a time *τ* is plotted in logarithmic scale on the vertical axis, for 3 representative cells in the population. a) *(left)* Positive selection homogenizes conjugation rates for all cells in the population, which is equivalent to normalizing the number of conjugations in a time interval; *(right)* Negative selection reduces the number of long conjugations. By combining positive and negative selection it becomes possible to access conjugation lifetimes—a reliable indicator given by the slope in the graphs—by measuring the rate of conjugations of a given duration. b) *(left)* Long lived conjugations, lasting longer than *τ*_*act*_, trigger cellular activation. Self-configurations are tolerated because most conjugations are short lived (*τ* < *τ*_*act*_); *(middle)* in the presence of abnormal self-configurations, several cells are mildly activated; *(right)* in the presence of nonself ligands, only a few T cells interacting with nonself ligands are strongly activated. c) By using anergy and costimulation, the impact of cells that escaped education is reduced because cellular activation occurs only when several T cells produce long conjugations.

The cellular frustration framework assumes that conjugation lifetimes are reliable anomaly detection indicators that can be used to trigger effector functions. This poses a problem to the immune system, namely that of measuring conjugation lifetimes using cellular contacts. In [48] it was proposed that the immune system may have solved this problem using two combined mechanisms. Positive selection would homogenize the dynamics associating to all cells conjugation lifetime distributions with close normalization factors. This allows measuring conjugation lifetimes by measuring the rate of conjugations of a given duration [49] (see Fig 2a).

The other important issue concerns how frustration can be changed by ordering ILists. In [49] it was shown that conjugation lifetimes are inversely proportional to the probability of destabilizing a conjugate and consequently are related to how cells prioritize interactions. In a stationary regime it was derived that conjugation lifetimes *τ*_*ij*_ can be calculated according to:

$$\begin{array}{c} \tau_{ij}∼\frac{1}{\sum_{kp}D_{kpij}{\tilde{n}}_{kp}} \end{array}$$ (1)

In this expression, non-null *k* and *i* indices denote APCs, while non-null *p* and *j* indices denote T cells. Null indices are used to account for non-conjugated cells. *D*_*kpij*_ is an integer that accounts for the number of ways *kp* (a conjugate or a single cell) can destabilize *ij*. *D*_*kpij*_ is 0 if *ij* cannot be destabilized. Note that Expression (1) is only valid in the stationary regime. Hence, ${\tilde{n}}_{kp}$ is the stationary frequency of *kp* conjugates or of *k* or *p* single cells (in case *k* or *p* are null, respectively).

Estimating the stationary frequencies ${\tilde{n}}_{kp}$ can be difficult because APCs can present diverse information and also because their dynamical equations involve numerous feedbacks [49]. Therefore finding stationary ${\tilde{n}}_{kp}$ frequencies demands self-consistent solutions which can only be found numerically. Nevertheless, this equation can still help us building a deeper understanding of mechanisms leading to accurate and sensitive immune discrimination.

First note that Eq (1) suggests that conjugations involving cells of the same subtype are the most stable. Indeed, in that case, only the T cell may be destabilized as the APC is already interacting with a ligand that is ranked in the top position. Therefore, by considering simply the impact of non-conjugated cells in the population, Eq (1) shows that the conjugation lifetime becomes at least inversely proportional to the sum of frequencies of non-conjugated APCs of the opposite cell type displaying ligands ranked above the ligand the T cell is interacting with. By contrast, if the same T cell is conjugated to an APC of the opposite cell subtype, the conjugate can be destabilized by all non-conjugated T cells of the opposite cell subtype, plus a number of non-conjugated APCs displaying ligands ranked higher in the IList than the ligand the T cell is interacting with. Thus we can conclude that long lived conjugations are mainly produced by conjugations involving cells of the same subtype. This is indeed what we obtain in numerical simulations.

Given the importance of distinguishing whether ligands are displayed by APCs of the same or of the opposite cell subtypes, we denote by LSCS, Ligands of the Same Cell Subtype and by LOCS, Ligands of the Opposite Cell Subtype.

Expression (1) also suggests that ILists could be organized to render the dynamics frustrated and allow accurate self-nonself discrimination. In [49] it was proposed that this organization could be established by the negative selection education mechanisms in the adaptive immune system. During education APCs present self-ligands. The whole set of self-ligands presented by all APCs at a given time, constitutes a configuration of the system, which changes from time to time. Negative selection eliminates all T cells interacting with high affinity with ligands displayed by APCs and replaces them by new incoming T cells (with random ILists). Within the CFF, high affinity interactions are measured by conjugation lifetimes and consequently, T cells are eliminated because they establish stable conjugations with APCs of the same cell subtype displaying ligands that are ranked in the T cell IList top positions. Therefore and except for a small fraction, only T cells without ligands displayed by APCs of the same cell sub-type on top positions of their IList can survive negative selection. This is true for all ligands frequently displayed by APCs. However, nonself ligands were not displayed by APCs. Consequently they cannot have made any impact on the education process and therefore, T cells surviving negative selection can still have them ranked on top positions. This implies that if nonself ligands are displayed by APCs in the periphery, many T cells may establish stable conjugations with these APCs.

In [48] it was argued that the CFF offers an integrated and consistent understanding of how the immune system performs immune detection. In particular, it was shown that costimulation and anergy improve considerably the accuracy of nonself discrimination by reducing the impact of errors in negative selection. This agrees with the interpretation of experimental data [50]. The CFF proposes that anergy and costimulation guarantee that APCs are only activated if multiple different T cells – and not just a single cell which could have escaped proper negative selection – establish long conjugations with an APC. Anergy is also important to force the immune system to test a wide range of different T cell receptors.

Costimulation and anergy are two of the three signals [38] required to mount an immune reaction. Within the CFF the third signal, which is usually associated to cytokines delivered to the medium [51], naturally arises from the fact that immune reactions should only be mounted once the sum of activation signals over all cells exceeds a threshold.

Fig 2 summarizes the main ideas and consequences that make cellular frustration a consistent framework. In the next section we will discuss how cellular frustrated systems (CFSs) detect signals of anomalies not arising from nonself ligands.

## Abnormal Self detection in Cellular Frustrated Systems

Consider a conjugate with cells of the same cell subtype in a frustrated population. Then, as discussed in the previous section, the conjugate can only be destabilized by non-conjugated APCs displaying LOCS that are ranked in top positions in the T cell IList. If the number of these LOCS diminishes, the denominator in Eq (1) decreases, increasing the average conjugation lifetimes. Since in the CFF, long-lived conjugations trigger effector functions, this suggests that CFSs should be capable of detecting other signs of intrusion besides the presence of nonself ligands. In fact, we will show that CFSs can detect two other types of perturbations. One is the increase in the number of ligands that are only rarely presented. These ligands are not nonself, because they appear in self-configurations. However, they appear only rarely and in this work they can be seen as the ligands appearing on the tails of distribution functions.

The other type of perturbation CFSs can react to occurs when uncommon combinations of frequent ligands are absent. This is a much more complex type of information as it captures correlations in presentation patterns. This detection mode goes well beyond single ligand statistics.

In this work it is assumed that T cells sense only two types of signals from each APC. From a modelling perspective this is equivalent to assume that each T cell maps the information displayed by each APC onto only two different ligands, i.e., as if only each APC would present one of two possible ligands. According to our results, the best immune protection is achieved when one of these ligands is perceived frequently and the other appears only rarely. This perspective agrees with that of several authors [39, 52–54]. Therefore, from here-on we refer to the information perceived as delivered by frequent or rare ligands. Note however, that this mapping differs from one T cell to the next, and that different APCs display different information and therefore their information is mapped onto different ligands. Note that different metrics can be used to establish this mapping, depending on the modeling taken to establish how T cells read the information presented by APCs. For instance, some models could focus on the interaction of the T cell receptor and the peptide-MHC complex, while others on avidity effects(see [55]).

An intuitive picture can then summarize how abnormal-self detection is achieved in CFSs. First, negative selection selects which sub-sets of ligands can be ranked in a small fraction of T cells ILists, on top positions, to minimize the frequency of long lived conjugations. Since negative selection uses a common and progressively adjusted lifetime threshold to eliminate T cells, all T cells adjust together the number of ligands kept under surveillance and in this way correlate their responses. As a result, after negative selection each T cell continuously surveys the presence of the set of frequent ligands it has ranked on top positions in its IList and displayed by APCs of the opposite cell subtype. If any of them is absent, the probability of establishing long conjugations with APCs of the same cell subtype increases. The amplitude of these individual cell responses is larger, the larger the number of absent frequent ligands. Using the long conjugation lifetimes observed in self-configurations, cellular activation thresholds can be defined.

The immune system as a whole is activated when the sum of individual cells activation signals (e.g., in the form of cytokines concentration) exceeds a threshold. This collective signalling can be related to the third signal required for the immune system activation, and it is stronger, the larger the number of activated cells. Hence, abnormal self-detection is triggered depending on the number of frequent ligands missing and on the number of T cells having them ranked in top positions.

## Results

Two types of results are presented next. First, the several detection mechanisms used by CFSs will be identified. This is best addressed using a specifically designed case study. Afterwards it will be shown that CFSs can achieve state of art anomaly detection performances in more general settings.

### DinBs: a case study with data presented in blocks

**Fig 3:**
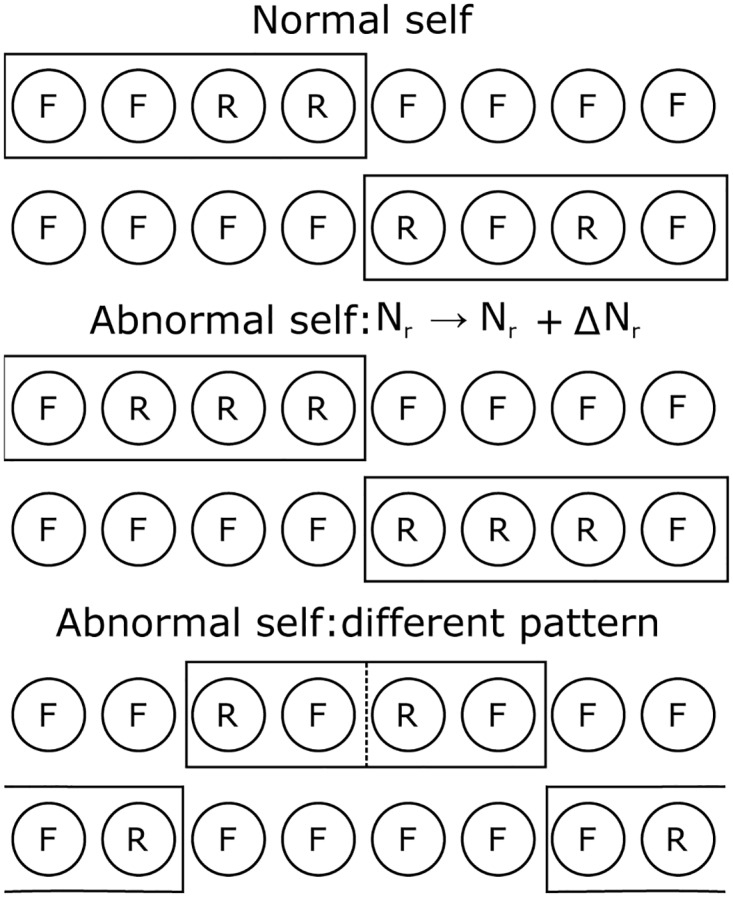
Illustration of the configurations displayed by APCs in the Data in Blocks (DinBs) case study. In circles and on each row are represented APCs of subtype I. Each APC can only present two ligands, represented either by an F or an R depending on whether they are frequently or rarely displayed. Different APCs display different ligands. Rectangular boxes delimit APCs that can randomly assigned to display rare ligands. For configurations with rectangular boxes with a dashed line, half of the number of rare ligands is displayed by APCs on one side of the box, and the other, on the other side. Configurations on the first four rows share the same presentation pattern but differ on the number of rare ligands displayed. Configurations corresponding to the last two rows, display sets of rare ligands that have never been displayed together when normal self-configurations were presented during education (maturation).

Consider a simplified version of the CFS model, in which *N*_*B*_ APCs belonging to first cell subtype either present a frequent or a rare ligand. In this example, it is assumed that all APCs present different ligands and all T cells use the same criteria to establish whether APCs display a rare or a frequent ligand (*N*_*B*_ = *N*/4). This is a special case of the more general model considered in this article, in which all T cells perceive differently the information presented by the several APCs. Since, in the models presented here, T cells map this information onto only 2 signals (or ligands), a frequently or a rarely displayed, this is also equivalent to assume that APCs present only either a frequent or a rare ligand (Fig 3).

In self-configurations either $N_{r}^{left}$ rare ligands are displayed by the first *N*_*B*_ APCs of the first cell subtype, or $N_{r}^{right}$ are displayed by the remaining APCs of the same subtype. The total number of rare ligands displayed in a configuration s is then $N_{r}\left(s\right)=N_{r}^{left}=N_{r}^{right}=N_{r}$.

Discrimination of two types of abnormal self-configurations will be tested. In the first case the number of rare ligands is increased: $N_{r}^{left}$ $=N_{r}^{right}$ = *N*_*r*_ + Δ*N*_*r*_. In the second case, the same number of rare ligands, *N*_*r*_, is displayed as in self-configurations. However, now half of them are presented by cells from the last *N*_*B*_/2 cells from the first block and the other half by cells from the first *N*_*B*_/2 cells from the second block of the first cell subtype. A representation of this data presentation scheme is shown in S1 Fig. An extension of this model considering that rare ligands could be presented by cells belonging to both cells subtypes would lead to similar results and therefore will not be considered here.

#### Maximally frustrated populations for CFSs with DinBs

Our initial analyses consider T cell populations with partially ordered ILists. These lists maximize frustration for all cells simultaneously, minimizing the longest conjugation lifetimes. To maximize frustration in the SCFSs with DinBs, ILists are organized according to the following strategy. First rank in top positions $N_{LOCS}^{top}$ frequent LOCSs; afterwards rank rare LSCSs; then rank frequent LSCSs; then rank rare LOCSs and finally rank the last frequent LOCSs. The specific way ligands are ranked in different T cells ILists also follows a specific order (see S2 Fig).

#### Mechanisms increasing the number of long lived conjugations

The number of stable conjugations can be increased in two ways: by increasing the probability of establishing stable conjugations by individual cells, or by increasing the number of cells establishing stable conjugations.

**Fig 4:**
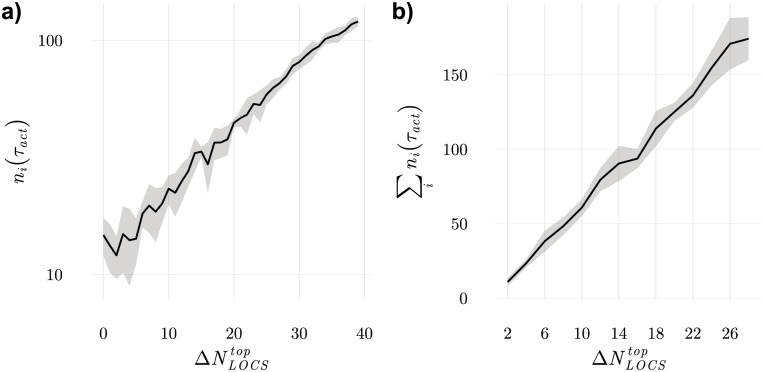
Cellular responses when ILists are specifically modified to analyse the impact of missing frequent ligands. a) the number of long lived conjugations grows exponentially when a growing number of frequent LOCS are removed from top positions in a T cell IList; b) the number of long lived conjugations grows linearly when a growing number of cells has a couple of frequent LOCS removed from their ILists top positions. In these results 100 realizations of systems with 96 cells were used.

To establish long conjugation rates, T cells have to establish stable interactions with LSCS. This happens when frequent LOCS ranked in top positions in T cells ILists are absent. Indeed, according to Eq (1), the largest conjugation rates increase with $\tau^{-1}\propto \sum {\tilde{n}}_{k\emptyset }$, where the sum over *k* runs over all APCs displaying LOCS on top positions in T cells ILists. One can then expect that the number of long lived conjugations established by a T cell with index *i* should be equal to $n_{i}^{0}\left(\tau_{act}\right)/n_{i}^{0}\left(0\right)=\exp\left(-\tau^{-1}\tau_{act}\right)=\exp\left(-\gamma N_{i,LOCS}^{top}\tau_{act}\right)$, where $N_{i,LOCS}^{top}$ denotes the number of LOCS on top positions in the *i*^*th*^ T cell IList, *γ* a proportionality constant and $n_{i}^{0}\left(0\right)$ a normalization constant. If $N_{i,LOCS}^{top}$ is decreased to $N_{i,LOCS}^{top}-\Delta N_{i,LOCS}^{top}$ then $n_{i}\left(\tau_{act}\right)/n_{i}\left(0\right)=n_{i}^{0}\left(\tau_{act}\right)\exp\left(-\gamma \Delta N_{i,LOCS}^{top}\tau_{act}\right)$. This is indeed confirmed in the results obtained in numerical experiments shown in Fig 4. In these experiments, all frequent LOCS are ranked on top positions in T cells ILists, except for those in one cell in which an increasing number of these ligands is moved to the lowest positions. The exponential dependency of the number of long lived conjugations on the number of removed ligands, $\Delta N_{LOCS}^{top}$, can then verified.

From these results we can conclude that T cells work as multiple correlation function evaluators responding whenever combinations of LOCS are absent. On this respect it should remarked that T cells are extremely efficient at performing this task since they evaluate multiple combinations at once. Another conclusion that can be drawn is that the established long conjugations are specific with respect to which LOCS are absent, but are not specific relatively to which LSCSs are actually producing the long lived conjugations. Indeed, changing the ranking of LSCSs in T cells ILists does not change the probability of establishing long lived conjugations. This is in strike contrast with nonself discrimination [48] in which case the APC that triggers the response displays the ligand recognized as nonself.

Instead of requiring that T cells are heavily perturbed, it is also possible to obtain the same increase in the number of long lived conjugations by increasing the number of T cells that engage, even if only mildly, in stable conjugations in the whole population. Then, in a first approximation equation (Eq (1)) predicts that if a number of T cell ILists is mildly perturbed, then long lived conjugation rates should increase linearly to their number. Likewise, the total number of long lived conjugations established in the whole population should increase linearly with the number of perturbed T cells. In Fig 4b we show that indeed, this is the case, irrespectively to which ligands disappear. This result shows that activation signals arising from multiple cells can add up to create proportionally reliable signals.

#### T cells detect increments on the number of displayed rare ligands

Now we show that T cells can detect deviations from normal presentations that are characterized by the presentation of a larger number of rare ligands than in self-configurations.

Denote by $\tau_{ij}^{0}\left(s\right)$ the conjugation lifetime performed by APC i∈A (A being the set of all APCs) and T cell j∈T (T being the set of all T cells) when a configuration $s\in S^{0}$ is presented ($S^{0}$ being the set of all configurations of self-ligands presented in the thymus). Denote by *τ*_*ij*_(*s*), the corresponding conjugation lifetime for configurations presented in the periphery. Then, in general, s∈S with $S^{0}\subset S$.

The first important observation is that the T cell population can discriminate, in principle, the presence of any rare ligand. To see this, consider first, that in self-configurations frequent LOCS had never been absent. Then, when the rare ligand is introduced in the maximally frustrated populations defined above with all frequent LOCS on top positions in T cells ILists (and all rare LOCS on the bottom), all T cells increase their largest conjugation lifetimes (with LSCSs) since the number of frequent LOCS is reduced when a rare ligand appears. Consequently, $\max_{i\in A}\left(\tau_{ij}\left(s^{\prime }\right)\right)>\max_{i\in A}\left(\tau_{ij}^{0}\left(s\right)\right),\forall s^{\prime }\in S,\forall s\in S^{0},\forall j\in T$. In principle, the same result should be obtained when only subsets of LOCS are on top positions in T cells ILists, provided all frequent LOCS appear in top positions in T cells ILists.

This was indeed confirmed in numerical simulations that counted the number of conjugations *n*_*j*_(*τ*_*act*_), lasting *τ*_*act*_ instants in a fixed time interval and established by a T cell with index *j*. In any configuration in which a rare ligand is introduced, there are always T cells for which $n_{j}\left(\tau_{act}\right)>\max\left(n_{j}^{0}\left(\tau_{act}\right)\right)$, where $n_{j}^{0}\left(\tau_{act}\right)$ is the number of long lived conjugations in normal configurations with no rare ligands displayed. Therefore discrimination is perfect in this extreme case.

The previous result requires that the number of rare ligands displayed in self-configurations is zero. This, however, is a serious drawback since it does not allow discriminating disturbances that are not linked to an increase in the number of rare ligands, but are due to different patterns of absence of frequent ligands (or, equivalently, different patterns of presentation of rare ligands).

To discriminate this type of disturbances it is crucial that frequent ligands are absent in self-configurations. In this case, however, an increase in the number of absent frequent ligands is not forcefully discriminated. This can be explained in a simple way by considering that one rare ligand is presented in self-configurations but, in the periphery, two rare ligands are presented instead. To tolerate the absence of one frequent ligand in any self-configurations, T cells should require that two LOCS ranked in top positions are absent in order to become activated. This, however, is not forcefully achieved if ILists only have subsets of frequent LOCS on top positions. In this case, triggering the system depends on the probability of activating at least one T cell. As we will show in the next section, this is the case of practical relevance, and therefore it should be studied in more detail.

Consider a maximally frustrated population with DinBs and with rare ligands presented only by APCs of one subtype in one of the two blocks. From the previous analysis, it follows that when the number of presented rare ligands is small, T cell activation should require the absence of two or more frequent LOCS ranked in top positions. The probability that *f* frequent LOCS are absent from top positions in T cells ILists is given by:

P(f)=(NLOCStop/2f)(NL/2-NLOCStop/2Nr-f)(NL/2Nr) (2)

where $N_{LOCS}^{top}/2$ is the number of frequent LOCS from one block ranked in top positions, *N*_*r*_ is the number of rare ligands presented in the configuration and *N*_*L*_/2 is the number of ligands from one block presented by APCs of subtype 1. The probability of activating a T cell is then *P*_*act*_ = 1 − *P*(0) − *P*(1). The activation of the whole system requires that a pre-defined number of T cells to be activated. This number depends on the false positive rate the system can safely accommodate. If one assumes that this false positive rate is denoted by *α*, then the number of T cells that must be activated to activate the whole system, *N*_*a*_, is the solution of the following equation:

∑n=0Na(NL/2n)Pactn(1-Pact)NL/2-n=1-α (3)

**Fig 5:**
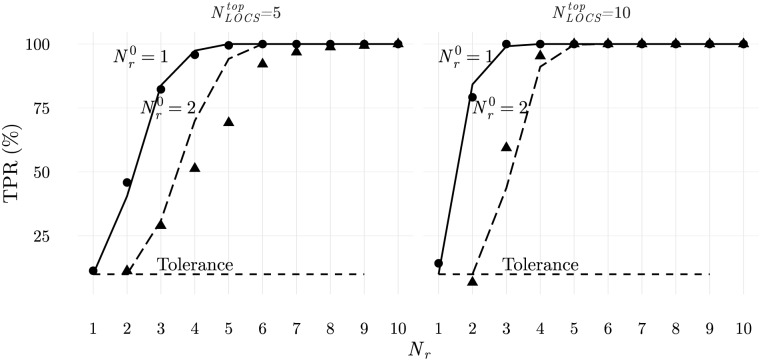
Average true positive rate when configurations with *N*_*r*_ rare ligands are presented to populations calibrated with $N_{r}^{0}=1$ or $N_{r}^{0}=2$ rare ligands. Results in dots and triangles are from numerical simulations where T cells have perfectly ordered ILists with $N_{LOCS}^{top}=5$ (left) or $N_{LOCS}^{top}=10$ (right) frequent *LOCS* in top positions from each block. Simulations used 96 cells of each type and 100 realizations. Solid and dashed lines are the predictions from the theoretical arguments described in the text. A false positive rate of 10% was assumed.

**Fig 6:**
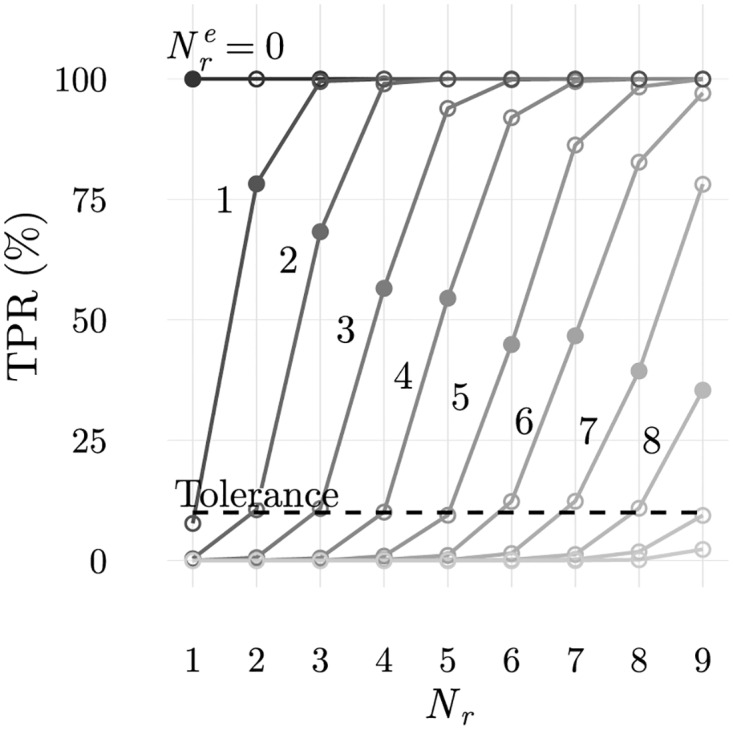
Average true positive rate when configurations with *N*_*r*_ rare ligands are presented to populations obtained by repertoire education and calibrated with several $N_{r}^{0}$ rare ligands. Highlighted with solid dots are results obtained when a single rare ligand is added to normal configurations. Larger numbers of rare ligands in normal configurations introduce noise reducing the capacity to detect perturbations.

Although the number of T cells of each subtype is *N*, by construction only half T cells have different IList in maximally frustrated systems for DinBs. The threshold value can be found by solving the fixed point recursive equation:

Na,n+1=Na,n-⌊λ∑n=0Na(NL2n)Pactn(1-Pact)NL2-n+α-1⌋ (4)

where *λ* is a parameter chosen to guarantee convergence (typically *λ* = 0.1) and ⌊⋅⌋ denotes the floor operation. The asymptotic solution of this equation gives the (threshold) number of cells above which the system is activated as a whole: $N_{a}^{thres}=N_{a,\infty }$. To evaluate the discrimination power when the number of rare ligands is increased, the probability of activating the whole system is calculated from:

Ξ(Nr0+δNr)=∑n=NathresNL/2(NL/2n)(Pact*)n(1-Pact*)NL/2-n (5)

where here $P_{act}^{*}$ is the probability of activating a T cell when $N_{r}=N_{r}^{0}+\delta N_{r}$, $N_{r}^{0}$ being the number of rare ligands presented in self-configurations. The results are presented in Fig 5, for $N_{r}^{0}=1,2$ and when *δN*_*r*_ is successively increased until full discrimination is achieved. Results obtained using the agent based numerical simulations with maximally frustrated populations are also depicted showing a good agreement. Furthermore, results from numerical simulations using the complete cellular frustration algorithm, with education of T cells ILists (discussed later in this article) are also shown, in Fig 6. The agreement among these results, confirm our interpretation of the mechanisms at play.

Two main conclusions can be drawn from these results. First, increasing successively the number of rare ligands increases the probability of discriminating the perturbation according to a logistic type of saturation growth curve. Indeed, consider the addition of a rare ligand, *N*_*r*_ → *N*_*r*_ + 1. Then, in a fraction of configurations the whole system is activated. If another ligand is introduced afterwards, a fraction of the formerly non activated configurations will have a similar probability of activating the system. If one assumes that this probability is roughly constant, then full discrimination is achieved and the logistic type of saturation growth curve displayed in Fig 5 is explained. More importantly, this result shows that the addition of an increasing number of rare ligands can eventually be always discriminated.

The second conclusion that is worth discussing is that the larger $N_{r}^{0}$ the harder it becomes to detect the addition of a rare ligand when $N_{r}^{0}$ is increased to $N_{r}^{0}+1$ ($N_{r}^{0}\to N_{r}^{0}+1$). Understanding this effect is important because it highlights the trade-off between tolerance and immunity. Consider the set of all self-configurations with $N_{r}^{0}$ rare LOCS (see S3 Fig). A large fraction of these configurations, 1 − *α*, has to be tolerated, while the remaining produce false positive activations.

Next consider the set of configurations with $N_{r}^{0}+1$ rare LOCS. To each configuration with $N_{r}^{0}$ rare ligands, $N/2-N_{r}^{0}$ configurations (*N*/2 being the number of subtype I APCs) can be constructed with $N_{r}^{0}+1$ rare ligands. Clearly, the fraction *α* of activated configurations with $N_{r}^{0}$ rare ligands, will also be activated when an extra rare ligand is added. Furthermore, a set of other configurations will also become activated, totally a fraction *δ* of activated configurations, corresponding to the fraction of true positives. Assume that *α* < *δ*, i.e., there is discrimination.

Next consider what happens if the number of rare ligands in self-configurations is $N_{r}^{0}+1$, and the population should detect the variation $N_{r}^{0}+1\to N_{r}^{0}+2$. From the fraction *δ* of activated configurations in the previous case, only a fraction *α* of the most activated configurations will activate the system, i.e., activation thresholds are now more demanding. Therefore, the set of configurations with $N_{r}^{0}+1$ rare ligands can be divided in 3 subsets: the most activated corresponding to the false positives when $N_{r}^{0}+1\to N_{r}^{0}+2$; the least activated, corresponding to false negatives when $N_{r}^{0}\to N_{r}^{0}+1$ (configurations not activated and with $N_{r}^{0}+1$ rare ligands); and the remaining activated configurations when $N_{r}^{0}\to N_{r}^{0}+1$. From this analysis it becomes clear that the discrimination power decreases when $N_{r}^{0}+1\to N_{r}^{0}+2$ relatively to $N_{r}^{0}\to N_{r}^{0}+1$, because the probability of creating activated configurations with $N_{r}^{0}+2$ rare ligands from the least activated configurations with $N_{r}^{0}+1$ rare ligands is smaller than the probability of generating these configurations with the intermediately activated configurations with $N_{r}^{0}+1$ rare ligands. Therefore, very general arguments explain the behaviour observed in the results in Fig 6.

In general, those configurations that previously activated the system, are now more likely to trigger the immune system when $N_{r}^{0}+2$ rare LOCS are displayed. This is because these configurations already have a larger number of activated cells, and therefore the number of T cells that should also become activated is smaller, or because there is a higher probability of further removing a frequent LOCS from the top of an activated T cell IList. However now, since thresholds are more demanding, only for a fraction of configurations the removed frequent LOCS can activate the immune system and consequently it should be expected a smaller discrimination power.

In simpler terms it could be stated that introduction of rare LOCS introduces noise which makes discrimination harder. However, in the next section it will be shown that this allows detection of other types of perturbations.

The previous mathematical approach assumed that populations displayed a small number of rare ligands in the thymus. In this limit, activation was controlled by the number of T cells sensing the absence of 2 frequent LOCS in tops positions. For self-configurations with a larger number of absent frequent LOCS, the threshold controlling the system’s activation may impose that a combination of T cells sense the absence of a different number of frequent LOCS (e.g., a fraction sense the absence of 2 while another fraction the absence of 3 frequent LOCS) since the system’s response aggregates the responses of all individual cells. The CFF proposes a set of immunologically plausible mechanisms to make this adaptive selection of criteria automatic. Clearly, the immune system, and agent based simulations in particular, have the advantage of being able to tune thresholds to adapt to complex presentation patterns.

#### T cells can sense contextual information

A more challenging detection capability consists in detecting disturbances that are not due to an increase in the number of rare ligands. Instead, it is the combination of ligands that determines whether a configuration is normal or abnormal. In this case the information is contextual. Within the CFF, T cells can still respond to this type of disturbances since responses depend on the combinations of absent frequent LOCS. However, a number of conditions have to be met. Firstly, the mapping into rare and frequent ligands should make rare ligands sufficiently frequent in order to provide information of presentation patterns. Secondly, frequent LOCS should not all be ranked on top positions. If this would be the case, then the number of absent frequent LOCS would not change as their overall number does not necessarily increase. Finally, and for the same reason, the system’s response should only receive contributions from cells sensing the absence of frequent LOCS as if all cells contributed their responses could cancel out. This naturally shows why single cell activation thresholds are needed.

Now we apply the previous mathematical analysis on maximally frustrated populations in the DinBs case study. To guarantee T cell’s tolerance towards the two types of normal configurations, T cells ILists should have on top positions the same number of frequent LOCS displayed by APCs of the two blocks. In this way, it is guaranteed that any T cell has at least $N_{LOCS}^{top}/2$ frequent LOCS on top positions. This number increases to $N_{LOCS}^{top}-N_{R}$ if $N_{r}<N_{LOCS}^{top}/2$.

Consider a specific example where $N_{LOCS}^{top}=N_{L}/4$, i.e., each T cell has on top positions half of the whole set of frequent LOCS of each block of data. In abnormal configurations, the same number of rare ligands is presented, but now half is presented on a block on the right and the other half on a block on the left (see Fig 3). The minimum number of frequent LOCS in ILists top positions is $N_{LOCS}^{top}-N_{R}$, but now this value has 0 as lower bound – see S4 Fig. Therefore, some cells sense the absence of a considerable larger number of frequent LOCS and will respond to the contextual change.

Consider that only two rare ligands are presented, both in normal and abnormal configurations. In this case, all steps taken before to find the activation threshold, $N_{a}^{thres}$ are still valid since normal configurations are the same. What differs is the population response to abnormal configurations. Since T cells should not have all frequent LOCS on top positions in their ILists, they sense the presence of frequent LOCS differently.

By construction, when $N_{LOCS}^{top}=N_{L}/4$, there are 2 ILists (identical) with *N*_*c*_ frequent LOCS in the central block (half on the left and the other half on the right) and $N_{LOCS}^{top}/2-N_{c}$ frequent LOCS outside. Since one rare LOCS is presented on each side, the probability that 1 frequent LOCS is missing from top positions in an IList is:

p1=(Nc/21)(NL/4-Nc/20)(NL/41)=2NcNL (6)

The probability that a T cell with *N*_*c*_ frequent LOCS in the central block, misses 2 frequent LOCS (one on each side) is:

$$\begin{array}{c} P\left(2,N_{c}\right)=p_{1}^{2}=4\frac{N_{c}^{2}}{N_{L}^{2}} \end{array}$$ (7)

Finding the probability of having more than $N_{a}^{thres}$ cells with 2 frequent LOCS missing is harder to calculate now than in the previous case. It amounts to calculate a convolution with $N_{a}^{thres}$ probability distributions. We computed this numerically by using a Monte Carlo approach. For a system with 48 APCs of each subtype (*N*_*L*_ = *N*/2 = 48) and when $N_{LOCS}^{top}=24$, we obtained $N_{a}^{thres}=9$ and Ξ_*context*_(*N*_*r*_ = 2) = 13.5% for *α* = 0.1.

**Fig 7:**
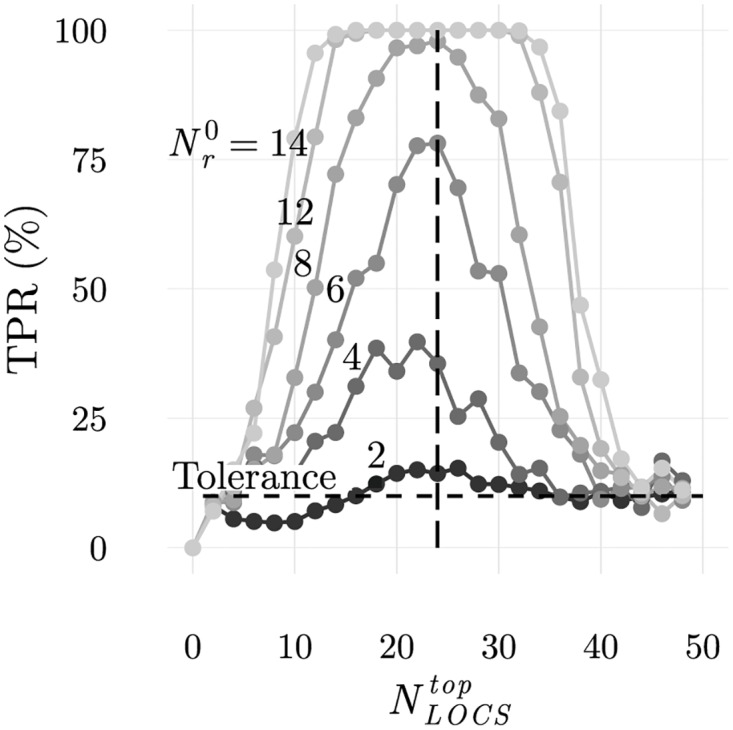
Average true positive rate for simulations for context dependent (abnormal self) discrimination. In the simulations considered for these results, both normal and abnormal configurations displayed the same number of rare ligands, although in different patterns as shown in Fig 3. Averages accounted 100 realizations and a false positive rate of 10%. Populations with 96 T cells and partially ordered ILists with $N_{LOCS}^{top}$ were used. From these results it is clear that the best discrimination is achieved for partially ordered ILists that maximize the number of (potentially) absent frequent LOCS in top positions. In the present case this number is 24, corresponding to the size of an ILists with all LOCS from the block presented in the abnormal self configuration. It is also clear that the larger the number of rare ligands displayed, or equivalently, the larger the number of absent frequent LOCS, the higher is the discrimination.

This agrees with the average true positive rate of 14.1% obtained after simulating 100 populations using the cellular frustration dynamics on populations with maximally frustrated ILists and shown in Fig 7. There, detection rates obtained when the number of frequent LOCS in top positions is varied are also shown.

Two important conclusions can be drawn from these results. Firstly, the number of frequent LOCS that should be ranked in top positions to maximize discrimination depend on the data presented. In this case rare ligands were presented in blocks with 24 APCs of the first subtype, and the maximum number of different ligands between normal and abnormal configurations was 24. As a result, the number of frequent LOCS that should be ranked in top positions to maximize discrimination was also 24. This shows that the immune system should find ways to automatically adapt to the information presented, ranking only an adequate number of frequent LOCS in top positions. This, of course contrasts with what was required to obtain the best results if the immune system was only concerned with detecting an increase in the number of displayed rare ligands. Therefore a trade-off exists as highlighted by these results.

Secondly, even if there are 24 frequent LOCS ranked in top positions (results along the dashed vertical line in Fig 7, the number of absent ligands is important to achieve the highest discrimination. In Fig 7, a minimum number, higher than 16, is required to obtain a 100% true positive rate. Increasing beyond this number, does not improve discrimination of contextual information, but it could be prejudicial for discriminating increments in the number of displayed rare ligands.

**Fig 8:**
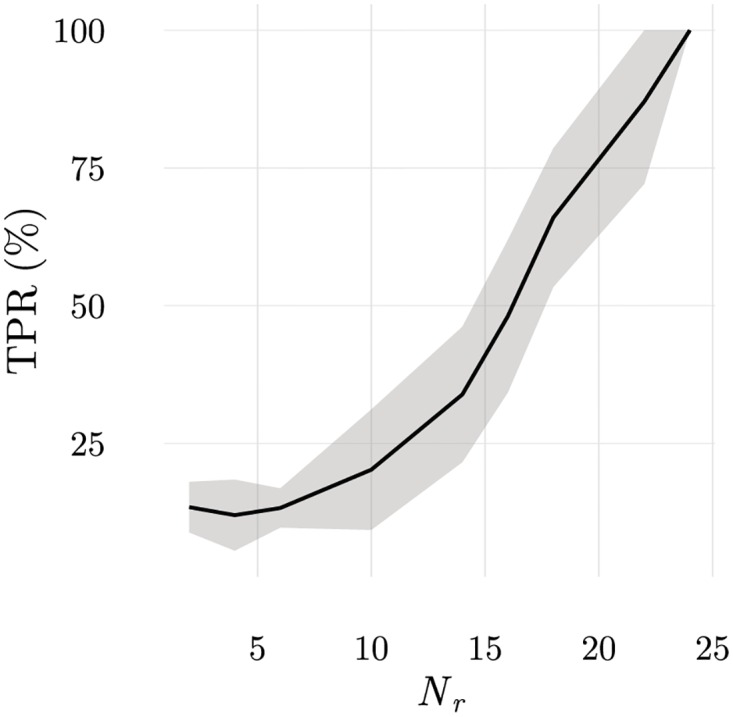
Abnormal self discrimination for populations with educated ILists when the number of rare ligands displayed is varied. Increasing the number of rare ligands in self and abnormal self-configurations increases discrimination. This result agrees with the theoretical arguments developed under the more restrictive conditions of Fig 7. In particular, it shows that context information can be perfectly discriminated even when ILists are ordered by negative selection.

In Fig 8 the true positive rate obtained with populations of T cells educated following negative selection, are also shown. These results show that the mechanisms of negative selection, which will be analysed more thoroughly next, also demonstrate that discrimination of contextual information is possible and improves with the number of rare ligands displayed. Results obtained with negatively selected populations lead however to poorer performances, which is expectable since maximally frustrated populations had been designed to obtain the best performances.

#### Negative selection is heterogeneous and selects T cells with the largest number of LOCS on top positions

**Fig 9:**
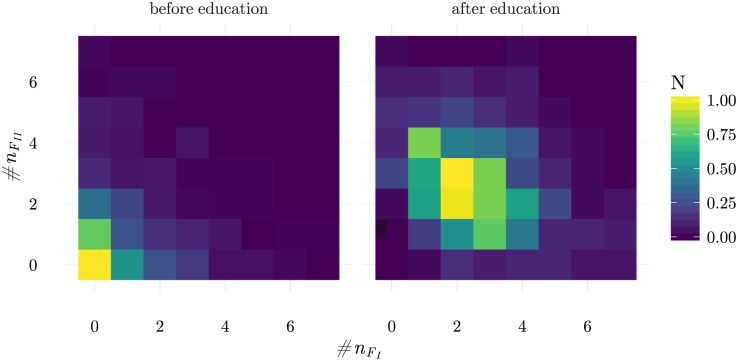
Frequency of the number of frequent LOCS of each block (I and II) on ILists above the highest ranked LSCS, before and after education (maturation). These results were obtained considering self-configurations with *N*_*r*_ = 6 (see Methods for other remaining simulation parameters). These results show that repertoire education balances the number of frequent LOCS of each block on ILists top positions, guaranteeing that a minimum number of frequent LOCS is always present in self-configurations.

The previous analysis provided two important results. The first was that contextual discrimination is possible and, in certain cases, even perfect. The second was that ILists should have on top positions a balanced number of ligands of each block so that there is always a minimum number of frequent LOCS present in any self-configuration. The important issue that should be addressed next consists in understanding how ILists organization and the number of LOCS in top positions can be chosen autonomously. In the adaptive immune system this is achieved in the thymus through repertoire education mechanisms. Within the CFF, negative selection operates by eliminating T cells producing the longest conjugation lifetimes. Since the longest conjugations occur for those cells sensing the absence of the largest number of frequent LOCS, negative selection should select T cells that rarely have less than a (threshold) number of frequent LOCS absent. When applied to the DinBs case study this is indeed what is observed, as shown in Fig 9. Therefore, negative selection produces the ordering required for contextual discrimination, as discussed in the previous section.

**Fig 10:**
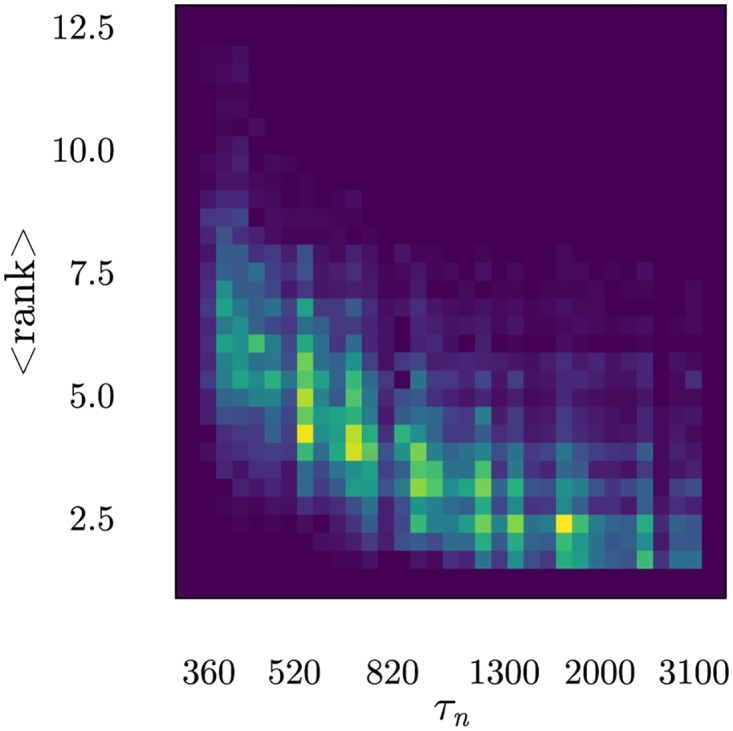
Two dimensional histogram for the mean rank of the first ranked LSCS in each IList as a function of the maximum conjugation lifetime *τ*_*n*_ in the population along repertoire education. It is clear from these results that LSCSs occupy progressively positions more on the bottom of ILists. For this figure 10 repetitions of the education of 120 populations with 96 T cells were considered. Self-configurations had *N*_*r*_ = 6.

Fig 10 shows two other important points. Firstly, when the maximum conjugation lifetime decreases, the average position of the LSCS ranked in the highest position in T cell ILists lowers, i.e., more LOCS are ranked in top positions. Secondly, the T cell population is considerably heterogeneous with the number of LOCS in top positions ranging from less than 5 to up to 10. This happens in spite of the fact that all T cells are equivalent in terms of their ILists organization. In this case, however, stochastic fluctuations are enough to break down this idealized equivalence. In practical applications, other sources of variation exist since not all APCs present the same information and also because, when limited connectivity is imposed on T cells ILists, T cells do not interact with all APCs.

#### Negative selection pushes frequent and rare LOCS at different rates

**Fig 11:**
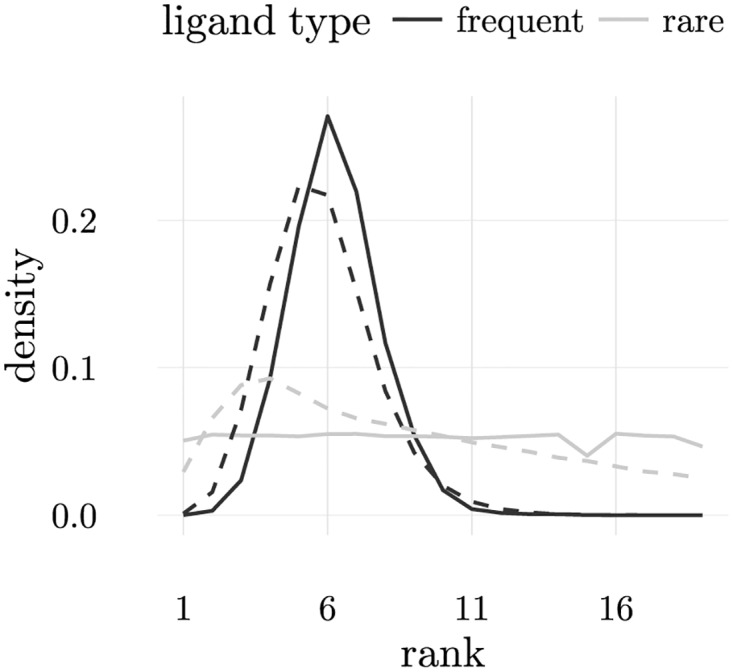
Distribution of the position occupied by frequent and rare LSCSs (dark and light coloured lines) after repertoire education for configurations with *N*_*r*_ = 0 (solid lines) and *N*_*r*_ = 6 (dashed lines). It is clear from this figure that frequent LSCSs tend to occupy lower positions than rare ligands. When *N*_*r*_ = 0, rare ligands are uniformly distributed in ILists, since they do not appear during repertoire education. When *N*_*r*_ = 6 the set of ligands appearing during education is larger making is more difficult to order ILists. As a result, in this case frequent LSCSs tend to occupy higher positions in ILists than frequent LSCSs in the *N*_*r*_ = 0 case.

The adaptive nature of T cell repertoire education can also be appreciated by noticing that frequent and rare LSCS tend to occupy different positions in T cells ILists after repertoire education. In particular, the highest ranked rare LSCS tend to be ranked higher than the highest rank frequent LSCS. This happens because negative selection operates more frequently on frequent LSCSs than on rare LSCSs. The paradigmatic example happens in the extreme case in which rare LSCSs do not appear during education, in which case they are nonself. Then negative selection cannot have an impact on how they are ranked in ILists. This can be confirmed in Fig 11, which shows that nonself ligands are ranked uniformly, while frequent LSCS have higher probability of occupying positions away from the top. Rare ligands appear less frequently then frequent LSCSs and consequently they have higher probability of accumulating in higher positions in ILists than frequent LSCSs.

Another important observation concerns the impact of the number of ligands presented on the ordering of ILists after negative selection. The distributions of rare and frequent LSCSs are centred on higher positions than the distribution of frequent LSCSs when nonself ligands are displayed. In fact, in the first case, the number of ligands that have to be “educated” is higher than when nonself ligands are presented, in which case there is effectively only half of the number of ligands.

There are two sources of limitations that prevent reaching perfectly ordered ILists. The first is statistical: the probability of randomly drawing a perfectly ordered IList decreases exponentially with the list size. The second originates from the intricate frustrated dynamics. When a T cell establishes a long conjugation with an APC, it prevents other T cells from interacting with the same ligand. As a result some of these T cells can be eliminated by negative selection since they sense a higher number of absent LOCS to this indirect effect. Every time a T cell is eliminated by negative selection, it is replaced by another naïve cell with a random IList, and consequently, in the whole population there is a high probability that it may be eliminated again in another upcoming configuration. Furthermore, every time a T cell establishes stable conjugations with some LSCS it may send other T cells to education as a side effect, making it hard to reach a state where all T cells have a similar probability of maintaining tolerance.

#### Generalisation capabilities depend on the presentation frequency

**Fig 12:**
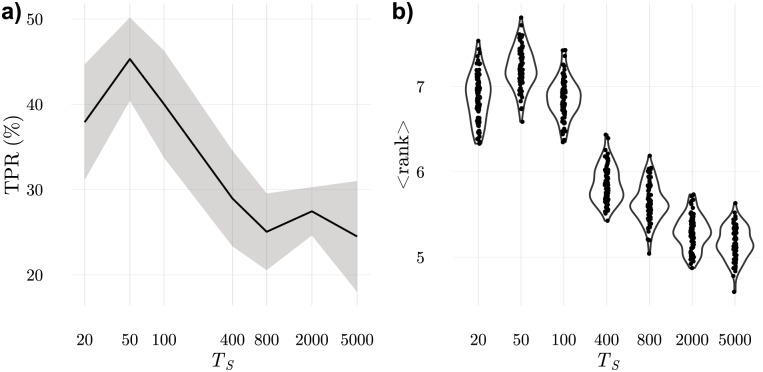
Generalisation capabilities are gained when samples are changed after every short time interval, *T*_*S*_. a) true positive rate for discrimination of configurations with an added rare ligand (*N*_*r*_ = 7) b) mean rank of the highest ranked LSCS in T cell ILists in the 200 samples used for education. These results show that ordering of ILists is best achieved when samples are changed every *T*_*s*_ = 50 iterations which also leads to the best discrimination. Since *τ*_*n*_—the maximum conjugation duration used to eliminate T cells by negative selection—is changed only when no cell is eliminated in the last *W*_*τ*_ = 10000 iterations, this forces ILists to be consistent with the last 10000/50 = 200 samples.

Another factor contributing to the creation of fluctuations during education is the rate of change of the information presented by APCs (presentation frequency). In our simulations, the configuration of ligands presented by APCs changes every *T*_*S*_ iterations. *T*_*S*_ has an important effect in the ordering of ILists and in detection. For very large values of *T*_*S*_, education strictly avoids having LSCS on top positions in any IList. For small values of *T*_*S*_, to be eliminated during education, it is required that in two consecutive configurations a T cell lacks frequent LOCS on top positions in its IList. Therefore, LSCSs, and in particular, rare LSCSs can appear on top positions in some ILists because the probability that they appear in consecutive configurations is $p_{R}^{2}$ which is small ($p_{R}^{2}∼2\%$ when *p*_*R*_ = 15%). Consequently, when *T*_*S*_ is small there will be more LOCS in top positions, on average, even if there may be some rare LSCSs among them. This agrees with the sequence of results in Fig 12).

### Applications

In the previous sections it was shown that CFSs can perform elaborate discrimination tasks. Now we evaluate how these discrimination capabilities compare to the performance of well-known statistical tests. We end by considering even more complex scenarios for which one must resort to data mining algorithms.

#### Can the immune system perform a t-test?

Probably the best known and most widely used statistical test is the t-test. For this reason we questioned whether cellular systems could perform this test with comparable accuracy. To define self-configurations, ordered samples with 80 numbers were randomly drawn from a Gaussian distribution with an average of *μ*_*S*_ = 50 and standard deviation *σ* = 10. Abnormal self-configurations were obtained by drawing samples from Gaussian distributions with the same *σ*, but with *μ*_*NS*_ = 50 ± Δ (two sided tests). The *i*^*th*^ APC displayed the *i*^*th*^ number in the sample as a ligand. Therefore, the first APC always presented the smallest number while the last APC, the highest. T cells sense these ligands as R or F ligands, depending on whether the number falls inside or outside an interval where a fraction *v*_*j*_ of the numbers displayed by an APC in normal configurations lie. Here, *j* is the T cell index, and *v*_*j*_ < 50% since it corresponds to the frequency of rare ligands.

In this article, T cells define rare ligands intervals for all ligands either on the right or on the left tail. Their size is controlled by the discrimination parameter *v*_*j*_ which is drawn from a uniform distribution between 0 and *v*_*max*_. As a consequence, T cells with *v*_*j*_ ≈ 0 are only sensitive to nonself ligands.

**Fig 13:**
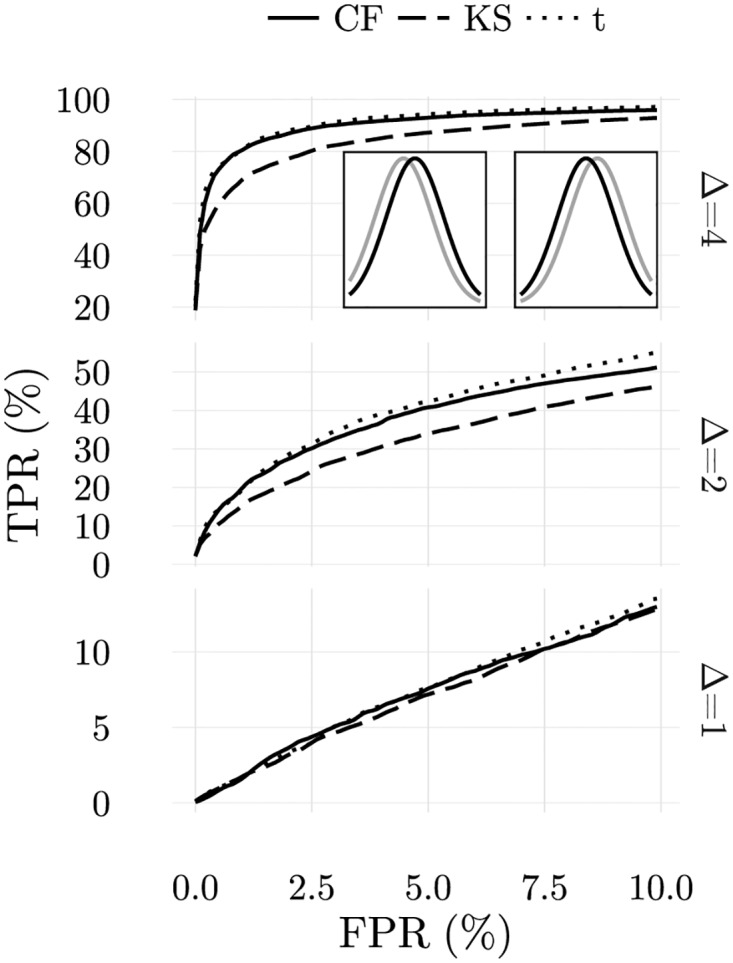
Comparative analysis of average ROC curves obtained in location tests using ordered samples with 80 elements drawn from normal distributions for the cellular frustration model and two-sided KS-test and t-test. Insets: comparison between gaussian distributions used to draw self-configurations (black) and abnormal configurations with deviations to either side (gray). Similar plots would be obtained for the distributions in the bottom examples. Abnormal-distributions are only slightly displaced. The t-test is the best estimator as demonstrated in the literature for this ideal case. However, cellular frustration models give very close results.

To assess how CFSs detect changes in *μ*_*S*_, the T cell population underwent negative selection, during which 1000 self-configurations were presented by APCs. Afterwards, APCs presented another 1000 self-configurations and 1000 abnormal self-configurations. In Fig 13, receiver operating characteristic curves (ROC curves) are presented for different changes in *μ*, with Δ = ±1, ±2, ±3. Comparison with the results that would be obtained with a t-test or a KS-test are also provided.

The best results are obtained with a t-test, as expected because data was prepared respecting its assumptions, and also because the t-test is a uniformly most powerful test for detecting deviations on the distribution mean [56–59]. By contrast and as expectable, the KS-test has the lowest performance since it is a simple nonparametric test. Results obtained with CFSs are, to a certain extent surprising since little assumptions were necessary on the type of information displayed by APCs and on the type of changes that could take place.

The CFF took three important assumptions in this example. The first was that the several elements in a sample could be ordered since all derived from a same Gaussian distribution. The same assumption is used in the KS test. The second assumption consisted in placing rare ligands in the tails. This guarantees that if the distribution deviates to either side the number of rare ligands present in the sample increases, and therefore this change has a high probability of being detected. Finally, the third assumption consisted in assuming that rare ligands in all APCs tend to appear on the same side of the distribution. This assumption makes sense because, given the ordering applied to the elements in the sample, a deviation in one tends to produce similar deviations in the next.

The three assumptions taken are important to obtain the best results, and they show if the immune system uses a cellular frustration strategy to perform discrimination, then evolution could have played a role to incorporate similar restrictions that allow reducing the space of potential disturbances. In the present problem, the first assumption makes sense given the nature of information presented. The second and third assumptions, could be relaxed if it would be important to contemplate a wider range of challenges. The same type of trade-offs distinguish parametric and nonparametric tests in statistics. In this sense, CFSs work as nonparametric tests, and are more accurate than KS tests, although computationally more involved. In fact, an interesting link exists between the KS approach and the CFF. In the KS test, the maximal deviation from a standard cumulative distribution determines whether the null-hypothesis should be abandoned. This decision crucially depends on sampling ordering. In the CFF the same deviations appear in the form of missed frequent ligands. The advantage of CFSs is to account to all deviations and furthermore to the presence of nonself ligands.

**Fig 14:**
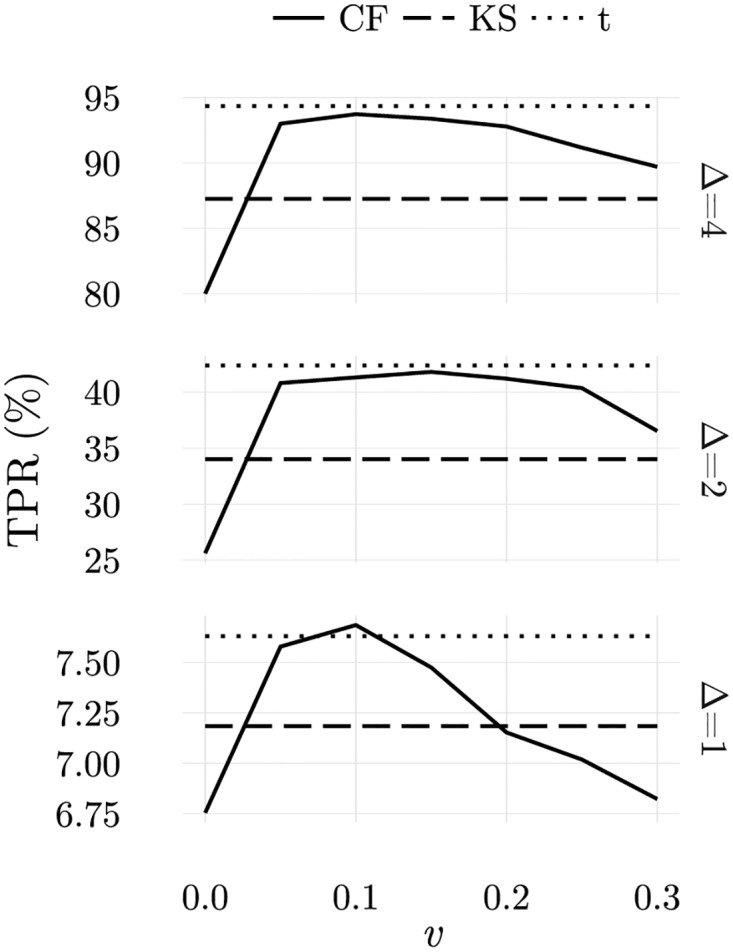
Average true positive rates obtained when the maximal probability of perceiving the information displayed by an APC as a rare signal (or ligand) is *v*_*max*_ for the three location tests conducted. Discrimination is best for *v*_*max*_ > 0 which means that discrimination is not exclusively due to the presence of nonself ligands. A 5% false positive rate was considered.

Another issue that should be addressed consists in knowing whether CFSs are robust if non-optimal parameters, such as *v*_*max*_, are used. This is particularly important because the optimal parameters typically depend on the challenge. In Fig 14 we show how results for a false positive rate of 5% change when *v*_*max*_ is changed. Clearly, when *v*_*max*_ is in the range [0.05, 0.15], results are consistently good for the different perturbations. This result is interesting because it shows that the best results are obtained when a fraction of cells perform abnormal-self detection, and not simply the detection of nonself ligands, i.e., outliers. From these results, we can conclude that the CFF contains the necessary mechanisms to tackle discrimination tasks conceptually equivalent to those solved by t-tests, an interesting result given the differences in the methods.

#### Can the immune system perform robust statistics?

An important area of research in statistics is concerned with improving the performance of statistical methods when the conditions required for their application are not met. CFSs are computationally more involved, so one could question if they offer robust solutions. We use the simple and paradigmatic example of the performance of t-tests in location tests when samples derive from a log-normal distribution. This case is challenging to the t-test because, even though, according to the central limit theorem, the distribution of the mean should converge to the normal distribution, the presence of heavy tails makes this convergence slow. This is particularly true for small samples. Consequently, the t-test can fail considerably.

**Fig 15:**
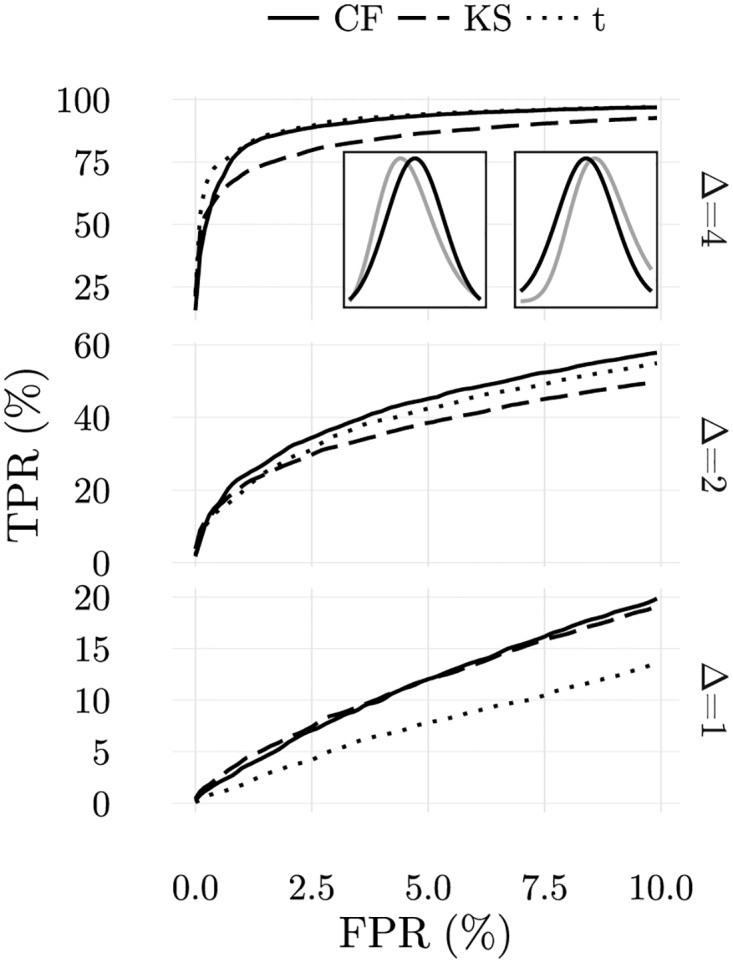
Comparative analysis of average ROC curves obtained in location tests using ordered samples with 80 elements drawn from lognormal distributions for the cellular frustration model and two-sided KS-test and t-test. Insets: comparison between the lognormal distributions used to draw self-configurations (black) and abnormal configurations with deviations to either side (gray). Cellular frustration models outperform the other statistical tests.

To test the accuracy of CFSs in this case, 1000 ordered samples with 80 numbers were drawn from log normal distributions with the same means and standard deviations as in the previous example. Furthermore, *v*_*max*_ = 0.05 was used and the same mapping into frequent and rare ligands was chosen. The ROC curves obtained are shown in Fig 15. CFSs demonstrate robustness over the range of perturbations considered.

#### Is the immune system a sophisticated data miner?

In more realistic scenarios, many pieces of information have to be accounted before triggering an immune response. Each of them can depend on different sources of variation and consequently a multivariate analysis is required. A simple way to mimic this more general scenario is to assume that in the previous examples samples are not ordered, i.e. each element in a sample is drawn from independent generators. Consider next the example in which, in self-configurations, each APC presents a ligand drawn from a Gaussian distribution centred at *μ*_*S*_ = 50 and with standard deviation *σ* = 10. Furthermore, in abnormal configurations *μ*_*S*_ is changed to *μ*_*NS*_ = 50 ± 4. These changes occur independently for each APC and in each configuration.

**Fig 16:**
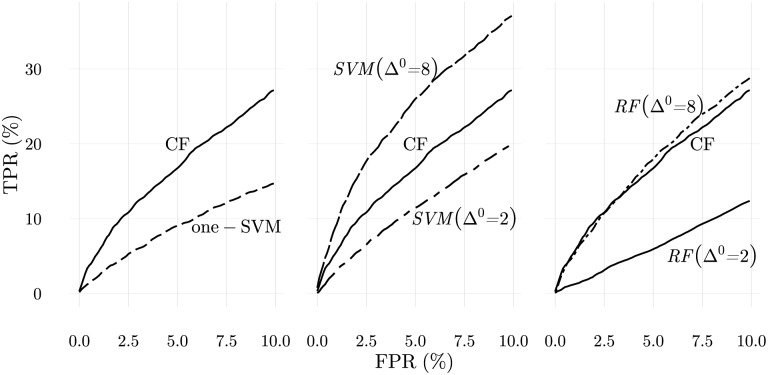
Comparison of the performance of the cellular frustration (CF) model with state of the art data mining algorithms. On the left the results obtained from CF model are compared with one-class SVM. The CF model clearly outperforms the data mining algorithm. In order to confirm that the results from the CF model are realistic, the same results are compared with support vector machines (center) and random forests (right) classification algorithms. Classification algorithms need to be trained with samples from abnormal configurations (drawn from Gaussians with mean deviated by Δ^0^), and consequently the comparison with the CF model, which uses only information from self-configurations, would be unfair. However, it shows that even with extra information, classification algorithms can fail to produce good results. This happens when the examples of abnormal configurations are not sufficiently distinct from those belonging to self-configurations. See text and Methods for more details.

To evaluate CFSs responses, the same strategy used to map APCs ligand information into rare or frequent ligands and to select the T cell repertoire is taken, and ROC curves calculated. To evaluate the performance of CFSs, a comparison with the best data mining techniques for this type of tasks was established (see Methods for more implementation details of these methods). The results are presented in Fig 16.

One-class SVMs, just like the CFSs defined here, only use self-configurations to infer what is normal and abnormal. Therefore, CFSs and one-class SVMs are methods that use the same information to accomplish the same task. The results shown in Fig 16 show that CFSs perform considerably well, outperforming one-class SVMs. Note nevertheless, that our goal here is not to argue that this may always be the case. In fact, SVMs solve an optimization problem and consequently datasets could be designed for which they provide the best solutions. Our goal is to demonstrate that it is not hard to find relevant problems for which CFSs are better, at least when standard approaches are used (i.e., using standard sets of parameters – see Methods).

Since one-class SVMs performances were so poor in comparison to CFSs, another set of numerical simulations was conducted to compare CFSs with classification methods, like two class SVMs and random forests (RFs). Classification methods require both, normal and abnormal configurations for training. Therefore, a direct comparison with CFSs is not straightforward. However, classification methods can indicate whether the information captured by CFSs and depicted in Fig 16 is indeed present, and how classification methods behave when the information provided for training does not perfectly match the information required for detection.

In Fig 16 a comparison of the performance of two class SVMs and random forests (RFs) with CFSs is presented. In these results, classification methods were trained with two sets of samples of the same size and generated from Gaussian distributions centred at *μ* = 50 ± Δ^0^. For self-configurations, Δ^0^ = 0, while for abnormal self-configurations Δ^0^ = 2 or Δ^0^ = 8. For detection, configurations centred around *μ* = 50 ± Δ, with Δ = 0 and Δ = 4, were used. These results show that if classification methods have access to abnormal configurations that do not strikingly contrast with self-configurations (Δ^0^ = 2), then discrimination of abnormal configurations centred around more distant positions (Δ^0^ = 4) can be problematic. In brief, classification methods work well only if distinctive examples are consistently presented. This, of course, can be problematic if only mildly abnormal self-configurations are available. In more mundane terms one could argue that classification methods are only good at predicting the occurrence of earthquakes if a strong earthquake of the same type has been felt before.

Finally, these results also show that the immune system can still improve its performance if it learns from examples of abnormal self-configurations. This, we know, the immune does, through clonal expansion mechanisms, a topic that we leave for a future publication.

## Conclusions and Perspectives

The results reported in this paper represent a landmark in the cellular frustration framework and its relevance to immunology because they clearly show that through simple cellular interactions the immune system could be activated with high precision.

The CFF proposed that cellular frustrated interactions could be crucial to build a system of cells in interaction whose dynamics would be extremely sensible to changes in population configurations [42]. This happened because generalized kinetic proofreading mechanisms were identified [42, 43, 60] making each T cell a context discriminator (or detector). Indeed, in [60] it was shown that in CFSs the dissociation constant that distinguishes alternative pathways before major signals are produced [61] is renormalized by factors related to cell frequencies in the population. The production of major signals was straightforwardly related to long-lived conjugations and it was proposed that the focus for understanding how the adaptive immune system works should be placed on which cells establish those long-lived conjugations.

In [49] it was shown that, despite the fact that CFSs are very sensitive to changes, a set of configurations could be defined to be tolerated. To select which configurations to tolerate, a new organization principle was proposed involving all interaction lists (ILists) in the population. The principle of maximal frustration suggested that ILists were selected to reduce conjugation lifetimes for all cells in the population and in this way taking into account the information in normal (self) configurations.

The previous results motivated a research program that had as main goal identifying the simplest frustrated system that could explain fundamental observations and simultaneously why the immune system is competent at protecting the host. In [48] it was shown that the simplest system could involve only APCs and T cells. This would be enough to explain why the immune system accurately detects nonself ligands in the periphery. However, to accomplish this, negative and positive selection would be required. Furthermore, costimulation and anergy would be necessary to improve the accuracy. These results are consistently accounted within the CFF scenario, as summarized in Fig 2.

By raising the question “Can the immune system perform a t-test?” this work calls attention to the fact that performing self-nonself discrimination could be largely insufficient to detect anomalies in the immune system. In statistics it is well known that the null hypothesis can be rejected even in the absence of outliers. It can be the presence of multiple small biases that signal an anomaly. Here we showed that CFSs could perform the same tasks as today’s most popular statistical and machine learning techniques, and with comparable, if not greater, accuracies. This can have profound immunological consequences since it was shown how the immune system can monitor simultaneously the presence of nonself ligands and the presence of combinations of frequently presented ligands.

The current results are part of a framework, a different mind setting to model cellular interactions and detect changes in the displayed information. These results suggest new directions of research, many of them naturally linked to immunology. Indeed, B cell activation was not considered, although the activation of B cells by T helper cells in lymph nodes [62, 63] may likely be explained using a similar modelling approach. Investigating how B cells are activated can also be extremely relevant because the considerable data that exists today [64, 65] makes it possible to confront theory and observation [66–68]. In this respect, it would be important to include the impact of effector functions like clonal expansion and the introduction of regulatory T cells to study how the immune response regulates homeostasis. To understand the immune system the CFF proposes finding the simplest model that is capable of performing the most accurate anomaly detection under plausible immunological conditions. For instance, in [49] we showed that three cell types (T cells, APCs and Tregs) could build a frustrated dynamics with detection capabilities. However, in [48] we showed that to achieve perfect self-nonself discrimination, Tregs were not required. Likewise, here we show that a model with only T cells and APCs is enough to explain how abnormal detection capabilities can be achieved. Therefore, so far, the CFF suggests that Tregs play no essential role for achieving good detection capabilities. This conclusion applies at least under static conditions. However, this conclusion may change if other requirements are imposed on how the information displayed evolves in time. This will be a matter of discussion in a future publication.

Another important conclusion resulting from this work concerns the role played by frequent and rare ligands for activation of the immune system. In [48] it was shown that nonself ligands—the rarest ligands possible—trigger specific cell activations, when these ligands appear in the system. By contrast, in abnormal-self discrimination, long-lived conjugations do not necessarily involve rare ligands and arise due to the absence of sets of frequent ligands. Therefore, the immune system monitors the two types of ligands in the system and in different ways.

Another point worth noting is that the results reported in this paper show how the immune system can be triggered to respond to changes in the displayed information. However, nothing is said concerning how responses can be set to resolve the causes of challenges. This is another topic to be discussed elsewhere and it will require an enlarged systemic approach to model the immune system.

How the real information displayed by APCs shapes the information contained in T cell ILists (i.e., T cell receptors) is a topic that should be further investigated taking into account the real aminoacid frequencies [53], details on recombinations events [69] or details on the interaction energies between APCs and T cell receptors [70].

Another issue that could be interesting exploring is the relation between CFF to other frustration models used in immunology [71–74] and possible connections to neuronal networks [75]. In particular, it would be interesting how the two classes of models compare at performing equivalent immune protection tasks.

Finally, the ideas explored in this article can be further developed in other directions. It would be interesting to consider whether the concept-drift idea [76] proposed to address anomaly detection in non-stationary datasets in artificially immune systems could also be useful, to explain the adaptive immune system. Another possible direction, would be to understand how danger theory ideas [24, 77] could be compatible with the CFFs. This work also suggests several ideas for research in machine learning, such as extending the current results to unsupervised learning [78] and even deep learning strategies in anomaly detection [79].

## Methods

### Agents Based Model

The agent based model used in this article can be summarized as follows. The model considers two cell types, APCs and T cells, each having two subtypes, *I* and *II*, with *N*/2 cells each. APCs favour interactions with T cells of the same subtype. Each APC presents a single ligand, *p*_*i*_. For an APC with index *i*, *p*_*i*_ is a real number between *i* and *i* + 1. Each T cell has a connectivity list with *K* different APCs with whom it can interact with. T cells map the information sensed on each APCs into only two possible signals (or ligands), *F*_*i*_ or *R*_*i*_, standing respectively for frequent or rare. Different specific mapping rules can be designed. In this article each T cell either senses rare ligands on the right or the left tail. For each T cell with index *j* a discrimination parameter *v*_*j*_ is drawn from a uniform distribution between 0 and *v*_*max*_. Designate $f_{i}^{0}\left(x\right)$ the frequency of occurrence of ligand *p*_*x*_ in self-configurations presented during education. Define left and right discrimination ligand thresholds, $p_{i}^{L}$ and $p_{i}^{R}$, such that:

$$\begin{array}{c} \int_{i}^{p_{i}^{L}}f_{i}^{0}\left(x\right)dx=v_{j},\;\int_{p_{i}^{R}}^{i+1}f_{i}^{0}\left(x\right)dx=v_{j} \end{array}$$ (8)

Then, a ligand *p*_*i*_ is mapped onto a rare ligand *R*_*i*_ if $p_{i}<p_{i}^{L}$ for T cells sensing rare ligands on the left tail, or $p_{i}>p_{i}^{R}$ if they sense them on the right tail. In the DinBs case study, all T cells used the same mapping and therefore each APC can be associated to a rare of frequent ligand. In this case, *p*_*i*_’s were selected so that the number of rare ligands in each configuration was fixed. In more general cases, *p*_*i*_ derive from the dataset.

Each T cell arranges *F*_*i*_ and *R*_*i*_ ligands in ordered interaction lists (ILists), prioritizing interactions with top ranked ligands (or signals). Modelling of cellular interactions assumes a discrete time dynamics where, at each time step, a randomly drawn cell is put in interaction with a cell from the other cell type and belonging to its connectivity list. A new conjugation is established whenever the two cells that are put in interaction, prioritize this interaction. In that case, if they were already conjugated, former conjugations are terminated and the duration of that conjugation is registered (see S1 Fig).

Negative selection in thymic repertoire education is modelled eliminating T cells that remain conjugate for a time longer than a threshold lifetime *τ*_*n*_ (see S1 Fig). A new cell is introduced in the population with the same connectivity list but with a randomly drawn IList. If after *W*_*τ*_ iterations (typically 10000 iterations) no cells exceeded the threshold time, then *τ*_*n*_ is updated to the largest conjugation time in the last *W*_*τ*_ iterations and the T cell population is registered. Every *T*_*s*_ iterations (typically 100 iterations), ligands presented by APCs present information from a different sample (configuration). When *τ*_*n*_ stops changing for at least 10^6^ iterations, education stops, the last recorded population is added to the T cell repertoire and education of a new T cell population is started, maintaining each T cell connectivity list but randomly drawing its IList.

After creating a repertoire of educated T cells, the population enters the calibration stage (see S1 Fig). This should correspond to the final stage of T cell repertoire education. At this stage cells engage in the same decision dynamics as before, except that anergy is introduced, so that T cells conjugated for a time longer than *τ*_*A*_ terminate their conjugations and are replaced by other cells with the same connectivity in the repertoire. In our results we used *τ*_*A*_ = 5. During calibration only self-information is presented. This is modelled by gathering information for presentation by APCs from the same set of samples that were used in the education stage. During calibration the decision dynamics is run for *W*_*c*_ iterations for each sample (typically *W*_*c*_ = 10^4^ iterations) and the number of conjugations lasting longer than an activation lifetime *τ*_*act*_, involving APC with index *i* when sample *s* is presented, $c_{i,s}^{0}\left(\tau_{act}\right)$, are registered. Defining the ordered vector $c_{i,(j)}^{0}\left(\tau_{act}\right)$, such that $c_{i,(j)}^{0}\left(\tau_{act}\right)\geq c_{i,(j+1)}^{0}\left(\tau_{act}\right)\forall j$, then an activation threshold is established by $n_{i}^{0}\left(\tau_{act}\right)=c_{i,(x)}^{0}\left(\tau_{act}\right)$ where $x=N_{s}^{c}\times f$, with $N_{s}^{c}$ the number of samples used during the calibration and *f* is a real number between 0 and 1. Typically we used *f* = 0.1, and hence the 10% biggest number of conjugations lasting a time larger than *τ*_*act*_ in a sample was considered. The activation reference time is chosen to be equal to the largest conjugation time in the calibration, i.e., *τ*_*act*_ = *τ*_*A*_.

To model cellular activation in lymph nodes the decision dynamics is run in the same conditions as in the calibration stage. To analyse the performance of the model in detecting anomalies, APCs display either information from a self-dataset, or from a nonself or abnormal-self dataset. Several examples are illustrated in the Results section. The cellular response to the information displayed by sample *s* is calculated according to:

$$\begin{array}{c} R_{s}=\sum_{i}\left(c_{i,s}\left(\tau_{act}\right)-n_{i}^{0}\left(\tau_{act}\right)\right)/c_{i,s}\left(0\right)\times \theta \left(c_{i,s}\left(\tau_{act}\right)-n_{i}^{0}\left(\tau_{act}\right)\right) \end{array}$$ (9)

where *θ* is the Heaviside function. Thus the cellular response sums the increments on the number of long conjugations relatively to the calibration stage. In this expression a subtle contribution from positive selection was taken. Instead of simulating the process explicitly, as in [48], we normalized the number of conjugations in the time interval *W*_*c*_ therefore accounting for the impact of positive selection as depicted in Fig 2.

To quantify the detection accuracy we compute the true positive rate for a fixed false positive rate, *FPR*. To achieve this we create and ordered vector of population responses to the $N_{s}^{d,s}$ samples in the detection stage displaying self-information, $R_{\left(i\right)}^{s}$, such that $R_{\left(i\right)}^{s}\geq R_{\left(i+1\right)}^{s}\forall i$ and find $R_{x}^{s}$, where $x=N_{s}^{d,s}\times FPR$. Then the true positive rate becomes $TPR=\#\left\{R_{s}^{ns}:R_{s}^{ns}>R_{x}^{s}\right\}/N_{s}^{d,ns}$, where $R_{s}^{ns}$ are the population responses to samples displaying nonself or abnormal self information and $N_{s}^{d,ns}$ is the number of nonself or abnormal-self samples presented during the detection stage. The true positive rate is thus equal to the fraction of samples displaying nonself or abnormal self information leading to cellular responses greater than $R_{x}^{s}$.

### Applications - dataset generation

Three synthetic datasets were generated to evaluate the performance of cellular frustrated systems (CFSs). All datasets are comprised of samples with 80 elements (*N* = 80).

The first two datasets were used for comparison with conventional statistical tests (t-student and KS-test). In the first case, self-samples were drawn from normal distributions with *μ*_*S*_ = 50 and *σ* = 10. Abnormal self-configurations were generated from normal distributions with the same standard deviation but with *μ*_*NS*_ = 50 ± Δ, where Δ = 1, 2 or 4 depending on the example. In the second case, samples were drawn from lognormal instead of normal distributions. Means and standard deviations were changed so that lognormal distributions maintained the same means and standard deviation.

The third data set was obtained as in the first dataset, however no ordering was applied. Furthermore, each element in abnormal-self samples could be drawn from a normal distributions deviated to either side. The example considered in this case used Δ = 4.

In each comparison, sets of samples were comprised of $N_{s}^{c}=1000$ self samples for training, $N_{s}^{d,s}=1000$ self samples for evaluation of the false positive rate, and $N_{s}^{d,ns}=1000$ to evaluate the true positive rate. When comparisons with data mining classification algorithms were considered, a supplementary set with 1000 abnormal-self samples with *μ*_*NS*_ = 50 + Δ^0^ were used for training. Two cases were studied: Δ^0^ = 2 or Δ^0^ = 8 (Fig 16).

### Applications - Statistical and data mining methods

Conventional two sided t and KS tests used the R implementation with known *σ*. Packages available in R, randomForests [80] and e1071 (libSVM) [81, 82] were used to apply two data-mining algorithms, respectively random forests and support vector machines. Two versions of support vector machines were considered, one-class SVM with a polynomial Kernel, and two-class SVMs with a RBF (radial basis function) Kernel. Both methods were applied using default packages parameters. This is particularly acceptable in case of anomaly detection, since knowledge of the abnormal class is not available. To compute average performances all tests were repeated ten times.

## Supporting Information

S1 Fig Numerical algorithm flowchart. (PDF)

S2 Fig Partially ordered interaction lists organization. (PDF)

S3 FigCompanion figure for the discussion of the decrease in the discrimination when a population is educated with $N_{r}^{0}$ rare ligands and $N_{r}^{0}+1$ rare ligands are presented for discrimination.(PDF)

S4 Fig Graph showing the evolution of the number of frequent LOCS in top positions in T cell ILists in the DinBs case study. (PDF)
