# Supplementary material for: Can the Immune System Perform a t-Test?
Source: PLoS One. 2017 Jan 3;12(1):e0169464. doi: 10.1371/journal.pone.0169464 (PMC5207702; doi:10.1371/journal.pone.0169464)
Supplement: S1 Fig — (PDF) [file pone.0169464.s001.pdf]

S1 Fig.-Numerical algorithm flowchart

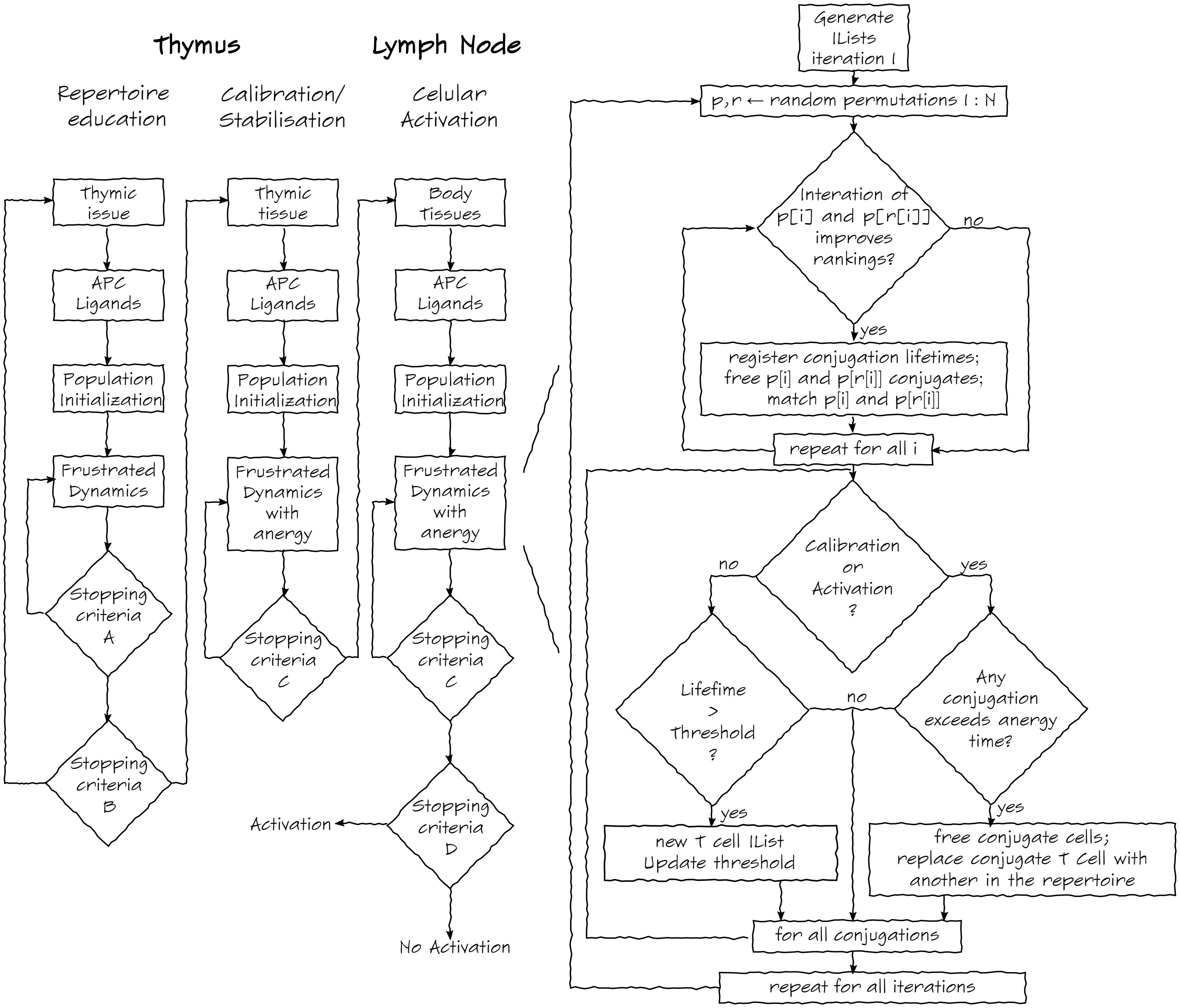

Figure 1: **Flowchart with the main steps in the numerical simulations used in this work.** In repertoire education negative selection is used to order T cell ILists until a stopping criteria A is met. This process is then repeated for several T cell populations (stopping criteria B). After repertoire education the system enters the calibration/stabilisation stage. The frustrated dynamics with anergy is run for W_c iterations (stopping criteria C), typically $W_c = 10^4$, to establish the characteristic frequency of conjugations lasting for a time τ. The same dynamics is run in the lymph node for cellular activation. However, now any samples can be presented (self or abnormal self). During cellular activation if a T cell exceeds the characteristic frequency of conjugations lasting for a time τ an immune response is mounted (stopping criteria D).
