# Supplementary material for: Can the Immune System Perform a t-Test?
Source: PLoS One. 2017 Jan 3;12(1):e0169464. doi: 10.1371/journal.pone.0169464 (PMC5207702; doi:10.1371/journal.pone.0169464)
Supplement: S2 Fig — (PDF) [file pone.0169464.s002.pdf]

S2 Fig.-Partially ordered interaction lists organization

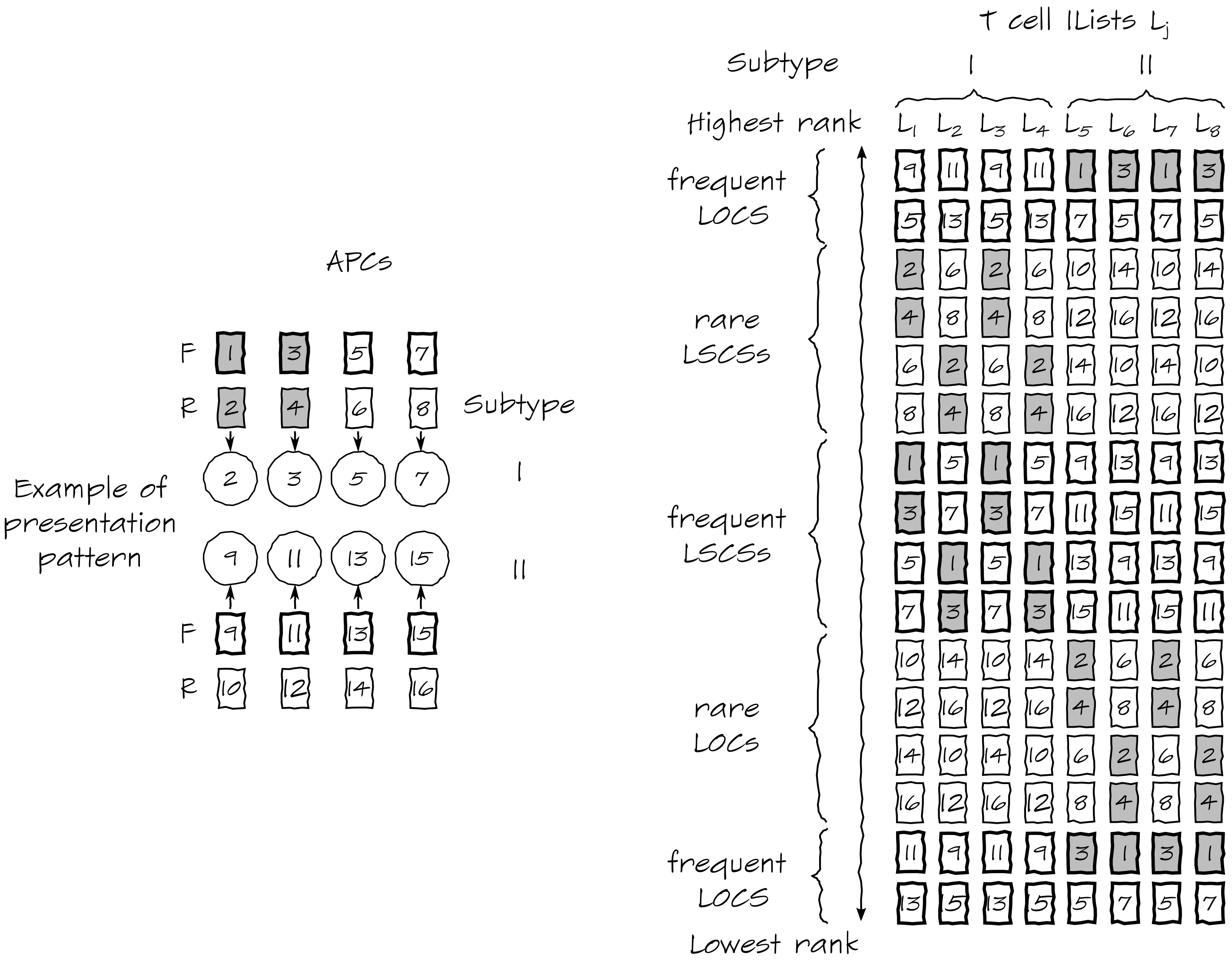

Figure 1: **Illustration of the generation scheme used to build T cell partially ordered ILists.** *Left*) Four APCs of two subtypes, I and II, can present either frequent or rare ligands (odd and even numbers, enclosed in darker or lighter boxes). In the configuration shown, the first APC presents a rare ligand (2) while the remaining APCs present frequent ligands. In this simple population with only four APCs of each subtype, the first two subtype I APCs belong to a same block (filled boxes). *Right*) Partially ordered T cell ILists with 2 frequent LOCS on top positions. Ligands are ranked sequentially. On top positions appear frequent LOCSs; then rare LSCSs; then frequent LSCSs; then rare LOCS; finally the remaining frequent LOCSs. Frequent LOCS use a different ordering scheme than the remaining ligands. Instead of being consecutively listed, frequent LOCSs are listed in pairs: ligand 7 is always listed after ligand 1; ligand 5 is always listed after ligand 3; ligand 15 is always listed after ligand 9; ligand 13 is always listed after ligand 11.
