# Supplementary material for: Can the Immune System Perform a t-Test?
Source: PLoS One. 2017 Jan 3;12(1):e0169464. doi: 10.1371/journal.pone.0169464 (PMC5207702; doi:10.1371/journal.pone.0169464)
Supplement: S3 Fig — (PDF) [file pone.0169464.s003.pdf]

S3 Fig.-Companion figure for the discussion of the decrease in the discrimination when a population is educated with N_r^0 rare ligands and N_r^0+1 rare ligands are presented for discrimination.

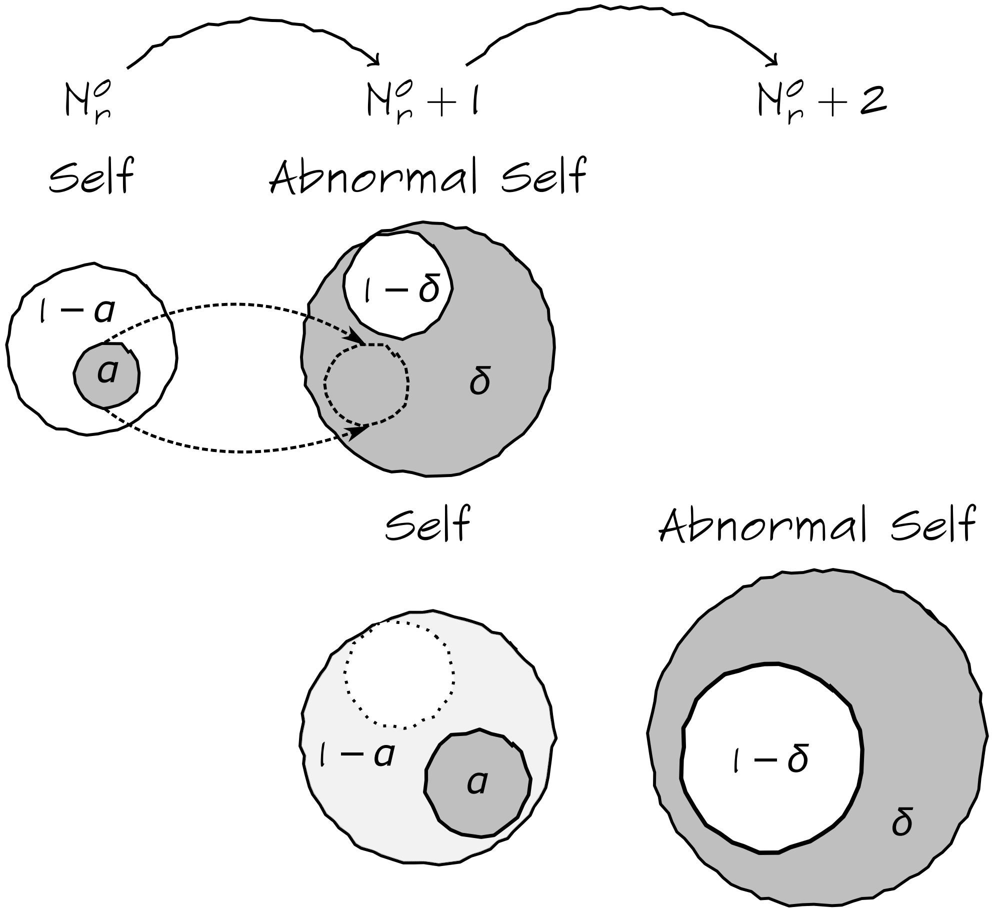

Figure 1: **Evolution of the number of activated configurations when the number of rare ligands is sequentially incremented.** On the top left, the set of self configurations with N_r^0 rare ligands are displayed. From these, a fraction α comprises those configurations leading to activation of T cells with the total highest magnitude. When an additional rare ligand the later configurations will remain activated and a fraction of the remaining $(1-\alpha)$ configurations will also lead to configurations with strong activations (indicated by dashed arrows and dashed circle). Overall, the fraction of the activated configurations will be δ. If one now considers that self configurations have N_r^0+1 rare ligands, since $\alpha < \delta$ only a subset of the later configurations − most activated of them − will lead to activated configurations. In this case, the magnitude required for activation is higher. Using the previous reasoning, that only a fraction of the least activated configurations become activated when an additional is displayed, then, when N_r^0+2 ligands are displayed, the most activated configurations with N_r^0+1 ligands will lead to activated configurations with N_r^0+2 ligands; A fraction of configurations with intermediate activation (represented in light grey) will also be activated; Only a very minor fraction of configurations in white will become activated when N_r^0+2 ligands are presented. Therefore, discrimination becomes poorer when the number of rare ligands displayed during education is higher and a rare is added for discrimination.
