# Supplementary material for: Can the Immune System Perform a t-Test?
Source: PLoS One. 2017 Jan 3;12(1):e0169464. doi: 10.1371/journal.pone.0169464 (PMC5207702; doi:10.1371/journal.pone.0169464)
Supplement: S4 Fig — (PDF) [file pone.0169464.s004.pdf]

S4 Fig.-Graph showing the evolution of the number of frequent LOCS in top positions in T cell ILists in the DinBs case study

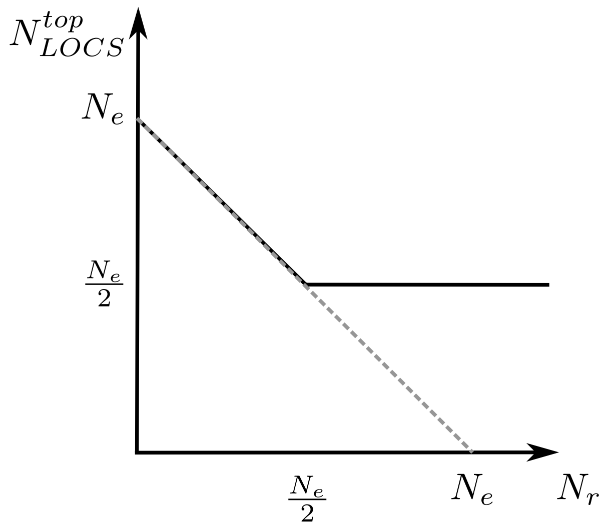

Figure 1: **Minimum number of frequent LOCS in a T cell IList for self (solid) and abnormal-self (dashed) configurations** Increasing the number of rare ligands has a different impact on the number of frequent LOCS in top positions in T cell ILists for self or abnormal-self configurations in the DinBs case study. The organization in T cell ILists guarantees that half of the top ligands belong to a block not displaying rare ligands in self configurations. However, for nonself configurations some ILists may have all frequent LOCSs on top positions absent.
